# Dose-Dependent Effects of Fermented Apple Pomace on Lactation Performance in Dairy Goats and Associated Antioxidant, Inflammatory, and Gut Microbiota Responses in Rats

**DOI:** 10.3390/foods15213770

**Published:** 2026-10-22

**Authors:** Jiongjie He, JianQiang Gao, Ling Wang, Shengyi Wang

**Affiliations:** Key Laboratory of New Animal Drug Project, Gansu Province, Key Laboratory of Veterinary Pharmaceutical Development, Ministry of Agriculture and Rural Affairs, Lanzhou Institute of Husbandry and Pharmaceutical Sciences of Chinese Academy of Agriculture Sciences, Lanzhou 730050, China; ajie7298@163.com (J.H.);

**Keywords:** fermented apple pomace, dairy goats, rats, dose-dependent, antioxidant, inflammation, gut microbiota, butyrate

## Abstract

Apple pomace, a byproduct of juice processing, requires valorization. This study evaluated the dose-dependent effects of fermented apple pomace (FAP) on growth and lactation performance in dairy goats and associated antioxidant, inflammatory, and gut microbiota responses in rats. Five FAP inclusion levels (0%, 5%, 10%, 15%, and 20%) were tested. In lactating dairy goats, 15% FAP was associated with the most favorable production and metabolic responses among the tested levels, including increased milk yield and average daily gain, reduced feed-to-milk ratio and somatic cell count, and lower blood urea nitrogen and ruminal NH_3_-N concentrations. However, nitrogen retention was not directly measured, and because FAP replaced corn and wheat bran, the diets were not isonitrogenous or isoenergetic; part of the responses may reflect changes in overall nutrient composition. In rats, 15% FAP was associated with activation of nuclear factor erythroid 2-related factor 2 (Nrf2) antioxidant signaling, suppression of Toll-like receptor 4 (TLR4)/nuclear factor kappa-light-chain-enhancer of activated B cells (NF-κB) inflammation, enrichment of butyrate-producing genera, and elevated cecal butyrate. These mechanistic findings are correlative and hypothesis-generating, requiring validation in ruminants. Collectively, 15% FAP was associated with the most favorable responses among the tested levels, but the mechanistic links between gut microbiota, butyrate, Nrf2/NF-κB signaling, and goat performance remain to be established.

## 1. Introduction

The juice processing industry in China generates millions of tons of apple pomace annually [1]. If improperly disposed of (e.g., open-air stacking or landfilling), this organic by-product, which is rich in fermentable sugars, pectin, polyphenols, and minerals, not only wastes biomass resources but also releases greenhouse gases and leachate due to its high moisture content, imposing significant environmental burdens [2]. The conversion of apple pomace, a non-grain biomass resource, into feed represents a pivotal direction for sustainable animal production and circular agriculture [3]. However, fresh apple pomace is characterized by high moisture (approximately 75–80%), relatively high fiber content (neutral detergent fiber of approximately 45% in the present study), and the presence of secondary compounds such as tannins, as well as pectin, which is primarily a structural and fermentable carbohydrate; these characteristics may affect feed intake and rumen fermentation in ruminants [4].

Solid-state fermentation (SSF) has been established as an effective approach to upgrade the nutritional quality of agro-industrial by-products [5,6]. Through microbial metabolism (e.g., by *Aspergillus*, *Saccharomyces*, or *Bacillus*), SSF degrades cellulose and hemicellulose, reduces anti-nutritional factors, and concurrently elevates crude protein content while enriching functional secondary metabolites including organic acids, polyphenols, and bioactive peptides [7,8]. The fermentation process used in the present study employed a composite starter culture of *Geotrichum candidum*, *Saccharomyces cerevisiae*, *Rhizopus* sp., *Bacillus subtilis*, and *Trichoderma viride*, with wheat bran, urea, and ammonium sulfate added to the medium. These microorganisms can degrade cellulose and hemicellulose, reduce anti-nutritional factors, and generate organic acids, polyphenols, and bioactive peptides. Urea and ammonium sulfate were added as nitrogen sources to support microbial growth. Therefore, FAP is a novel product whose composition is determined by both the fermentation process and the added ingredients. The observed effects may be attributed to the apple pomace itself, the fermentation process, or the added nutrients, and this should be considered when interpreting the results. Despite sporadic reports on the application of fermented apple pomace (FAP) in monogastric animals such as pigs and poultry [7], systematic investigations into its effects on ruminants—particularly dairy goats—remain remarkably scarce. In particular, there is a pronounced knowledge gap regarding rumen fermentation characteristics, systemic metabolic reprogramming, and the underlying immuno-regulatory and antioxidant molecular mechanisms mediated by FAP in ruminants.

Notably, the gut microbiome, as a “second genome” that integrates nutrient sensing and immune modulation, has been demonstrated to serve as a core driver in dietary intervention-mediated host health improvement [9]. However, whether FAP can reshape the ruminal and hindgut microbial community structure to generate specific bioactive metabolites, such as short-chain fatty acids (SCFAs), and consequently activate host defense systems via defined signaling cascades (e.g., the nuclear factor erythroid 2-related factor 2 (Nrf2)/nuclear factor kappa-light-chain-enhancer of activated B cells (NF-κB) axis), remains entirely unexplored. Moreover, the most favorable inclusion level of FAP in ruminant diets and the potential dose-dependent effects have not been systematically evaluated.

Given that goats are not amenable to fine-scale molecular and histological analyses, this study adopted a dual-model complementary strategy: lactating dairy goats were used to assess production performance, rumen fermentation, and systemic metabolism, while rats were employed to deeply dissect hepatic Nrf2/NF-κB signaling and gut microbiota–metabolite crosstalk. We hypothesized that FAP enriches butyrate-producing genera and elevates cecal butyrate production, and that butyrate, as a signaling molecule, activates Nrf2 nuclear translocation while suppressing the NF-κB inflammatory pathway, thereby systemically enhancing antioxidant capacity and immune homeostasis [10,11] and ultimately improving growth and lactation performance; moreover, this effect exhibited a dose-dependent pattern, with the most favorable responses observed at 15% FAP among the tested inclusion levels, whereas excess supplementation potentially elicited negative feedback.

Accordingly, the objectives of this study were: (1) to determine the inclusion level of FAP associated with the most favorable responses among the tested levels in dairy goat diets; and (2) to explore, using a complementary rat model, the potential involvement of the gut microbiota–butyrate–Nrf2/NF-κB axis, thereby providing a theoretical basis and industrial-scale parameters for the high-value utilization of apple pomace as a feed resource.

## 2. Materials and Methods

### 2.1. Preparation of Fermented Apple Pomace (FAP)

Fresh apple pomace was sterilized at 121 °C for 15 min and cooled to ambient temperature prior to use. Fermentation was performed using a composite solid-state starter culture consisting of *Geotrichum candidum*, *Saccharomyces cerevisiae*, *Rhizopus* sp., *Bacillus subtilis*, and *Trichoderma viride* at an optimized viable count ratio of 2:2:0.5:0.5:1 (obtained from the China Center of Industrial Culture Collection, Beijing, China). The fermentation medium was formulated on a mass fraction basis as follows: apple pomace 81.3%, wheat bran 15%, urea 1.5%, ammonium sulfate 2%, dipotassium hydrogen phosphate (K_2_HPO_4_·3H_2_O) 0.10%, and calcium carbonate (CaCO_3_) 0.10%. Urea was filter-sterilized (0.22 μm) and added aseptically after the medium had cooled. The mixed starter was inoculated at 10% (*v*/*w*), and the initial moisture content was adjusted to 60-65% with sterile water. After thorough mixing, the mixture was spread evenly to a layer thickness of 5 cm and subjected to solid-state fermentation at 31 °C for 72 h. Upon completion, the fermented product was oven-dried at 60 °C to constant weight, ground to pass through a 40-mesh sieve, and stored in sealed bags at 4 °C until further use. The pH, dry matter content, true protein, non-protein nitrogen, and residual urea/ammonium concentrations of the final FAP product were not determined in the present study; therefore, the contribution of residual non-protein nitrogen to the apparent increase in crude protein cannot be excluded.

### 2.2. Animals and Housing

Two independent animal experiments were conducted to evaluate the effects of FAP on production performance in dairy goats and the underlying molecular mechanisms in rats. All procedures were approved by the Institutional Animal Care and Use Committee of the Lanzhou Institute of Husbandry and Pharmaceutical Sciences, Chinese Academy of Agricultural Sciences (approval No. LIHPS-2018-022, 25 March 2018), and performed in accordance with the Regulations for the Administration of Laboratory Animals of the People’s Republic of China.

#### 2.2.1. Dairy Goat Trial

Thirty healthy lactating dairy goats (initial body weight 55.5 ± 3.2 kg, with similar body condition, parity, and lactation stage) were randomly assigned to five groups (*n* = 6 per group). Goats were tethered in individual stalls, fed twice daily (08:00 and 17:00), and had free access to water. The barn temperature was maintained at 22 ± 2 °C with relative humidity of 50–60%. The experiment lasted 60 days, including a 14-day adaptation period followed by a 46-day formal trial.

#### 2.2.2. Rat Trial

Fifty specific-pathogen-free (SPF) male Sprague-Dawley rats (8 weeks old, weighing 200–220 g) were acclimatized for 1 week and then randomly assigned to five groups (*n* = 10 per group) using a random number table. Male rats were used exclusively to avoid confounding effects of estrogen-induced fluctuations in oxidative stress and immune parameters. Rats were housed in barrier facilities under controlled conditions (22 ± 2 °C, 50% ± 10% relative humidity, 12 h light/dark cycle) with ad libitum access to diet and water. The trial lasted 4 weeks.

### 2.3. Experimental Diets and Grouping

**Figure 1:**
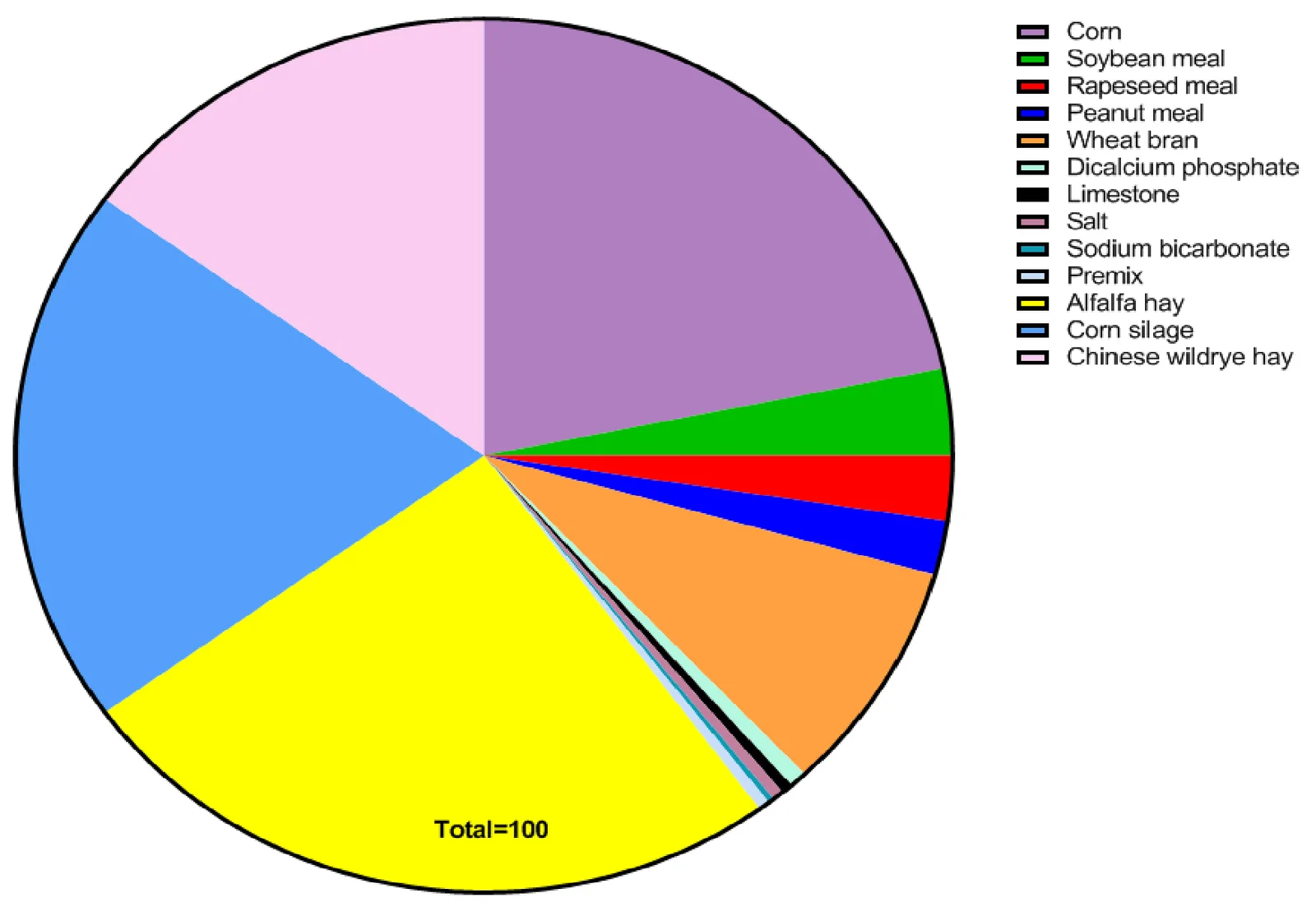
Composition of the basal diet (0% FAP) for lactating dairy goats (% of dry matter). The basal diet was formulated with a concentrate-to-forage ratio of 40:60 on a dry matter basis. Values are expressed as percentages of total dietary dry matter. The concentrate mixture included corn, soybean meal, rapeseed meal, peanut meal, wheat bran, dicalcium phosphate, limestone, salt, sodium bicarbonate, and premix; the forage portion comprised alfalfa hay, corn silage, and Chinese wildrye hay. FAP, fermented apple pomace.

For dairy goats, the basal diet was formulated on a dry matter (DM) basis with a concentrate-to-forage ratio of 40:60. The concentrate mixture, accounting for 40% of dietary DM, comprised corn, soybean meal, rapeseed meal, peanut meal, wheat bran, dicalcium phosphate, limestone, salt, sodium bicarbonate, and a vitamin–mineral premix. The forage portion, accounting for the remaining 60% of dietary DM, consisted of alfalfa hay (25%), corn silage (20%), and Chinese wildrye hay (15%). The composition of the basal diet is shown in Figure 1 and Table A1, and the composition of the vitamin–mineral premix is shown in Supplementary Table S1. FAP was included at 0%, 5%, 10%, 15%, or 20% of dietary DM by substituting equivalent proportions of corn and wheat bran within the concentrate mixture. Specifically, each 5% increment of FAP replaced 3.6% corn and 1.4% wheat bran on a dietary DM basis, while all other concentrate ingredients and the forage portion remained unchanged; consequently, the total concentrate proportion was maintained at 40% and the concentrate-to-forage ratio at 40:60 across all treatments. The graded FAP inclusion levels and the corresponding ingredient composition of the experimental diets are presented in Supplementary Table S2. All ingredients were mixed into a total mixed ration (TMR), which was prepared fresh daily.

For rats, the basal diet was formulated according to the AIN-93M purified diet. FAP was incorporated by weight-for-weight substitution for microcrystalline cellulose in the AIN-93M diet to maintain comparable total dietary fiber levels among groups. The exact formulation is provided in Supplementary Table S3. Because FAP replaced corn and wheat bran, the experimental diets differed in CP, NDF, ADF, EE, NFC, and Ca and were not isonitrogenous or isoenergetic. This should be considered when interpreting treatment effects.

#### 2.3.1. Lactation Performance Measurement

Feed offered and refused were recorded daily for each goat before the morning feeding (08:00), and dry matter intake (DMI) was calculated as (feed offered—feed refused) × dietary dry matter content (determined as described in Section 2.5). Milking was performed twice daily (06:00 and 18:00), and daily milk yield (kg/d) was recorded. On a fixed day each week (Monday), milk samples were collected in proportion to morning and evening milk yield, preserved with potassium dichromate, stored at 4 °C, and analyzed for milk fat (%), milk protein (%), and lactose (%) using a milk analyzer (Foss MilkoScan™ FT+, Foss, Hillerød, Denmark). Somatic cell count (SCC, ×10^3^ cells/mL) was determined using a Fossomatic™ 7 (Foss, Hillerød, Denmark). Body weight was measured on two consecutive days before the morning feeding at the start and end of the formal trial, and the average of the two measurements was used as the initial and final body weight to calculate average daily gain (ADG, g/d). The feed-to-milk ratio was calculated as DMI/milk yield (kg/kg). All milk samples were analyzed within 24 h of collection. Daily dry matter intake (DMI), daily milk yield, and weekly milk composition were recorded repeatedly during the experimental period. For statistical analysis, these repeated measurements were first averaged for each animal over the experimental period, and the animal was considered the experimental unit. Therefore, each animal contributed a single value for each variable. This approach avoided pseudoreplication and accounted for within-animal dependence.

#### 2.3.2. Blood Sampling

Goats: On day 46 (the final day of the formal trial), before the morning feeding, blood samples (10 mL) were collected from the jugular vein into serum separator tubes (BD, Franklin Lakes, NJ, USA). After clotting at room temperature for 30 min, serum was separated by centrifugation at 3000× *g* for 10 min, aliquoted, and stored at −80 °C for subsequent biochemical and hormonal analyses.

Rats: At the end of the experiment, rats were anesthetized by intraperitoneal injection of sodium pentobarbital (50 mg/kg body weight; Sigma-Aldrich, St. Louis, MO, USA) and then euthanized by exsanguination via the abdominal aorta. Serum separation and storage were performed as described for goats, for antioxidant and cytokine assays.

#### 2.3.3. Rumen Fluid and Tissue Sample Collection

Rumen fluid (goats): Immediately after blood collection, approximately 50 mL of rumen fluid was withdrawn via an esophageal tube, discarding the initial 20 mL to minimize salivary contamination. The fluid was filtered through four layers of gauze, and pH was measured immediately using a portable pH meter (Mettler Toledo, Columbus, OH, USA). For VFA and NH_3_-N determination, an aliquot was centrifuged at 10,000× *g* for 10 min at 4 °C, and the supernatant was acidified with 25% metaphosphoric acid (final concentration 2.5%, *w*/*v*) and stored at −80 °C.

Tissues and contents (rats): After exsanguination, the liver was rapidly excised, rinsed with ice-cold saline, minced, snap-frozen in liquid nitrogen, and stored at –80 °C for RT-qPCR and Western blot analyses. Cecal contents (for SCFA measurement) and fresh feces (for 16S rRNA sequencing) were aseptically collected, immediately frozen in liquid nitrogen, and stored at −80 °C.

### 2.4. Nutrient and Amino Acid Composition Analysis

Dry matter (DM, AOAC 930.15) was determined by oven-drying at 105 °C to constant weight. Crude protein (CP, AOAC 984.13) was measured by the Kjeldahl method (N × 6.25). Ether extract (EE, AOAC 920.39) was determined by Soxhlet extraction with petroleum ether. Ash (AOAC 942.05) was measured by incineration at 550 °C for 6 h. Neutral detergent fiber (NDF) and acid detergent fiber (ADF) were determined using a fiber analyzer (ANKOM Technology, Macedon, NY, USA) following the method of Van Soest et al. [12], with heat-stable α-amylase and sodium sulfite added during NDF analysis; both NDF and ADF values are expressed exclusive of residual ash. Calcium and phosphorus were analyzed according to AOAC 975.03 and AOAC 965.17, respectively, after wet digestion with HNO_3_-HClO_4_. Non-fibrous carbohydrates (NFC) were calculated on a dry matter basis according to NRC (2001) [13]: NFC (%) = 100 − (CP + NDF + EE + Ash). All nutrient values were expressed on a 100% dry matter basis. Amino acid profiles were determined using an amino acid analyzer (Hitachi, Tokyo, Japan) after acid hydrolysis with 6 M HCl at 110 °C for 24 h; sulfur-containing amino acids (methionine and cysteine) were oxidized with performic acid prior to hydrolysis, and tryptophan was determined separately after alkaline hydrolysis (4 M LiOH, 110 °C, 24 h).

### 2.5. Rumen Fermentation Parameters

Rumen fluid was centrifuged at 10,000× *g* for 10 min at 4 °C, and the supernatant was acidified with 25% metaphosphoric acid (final concentration 2.5%, *w*/*v*). pH, total volatile fatty acids (TVFA), and NH_3_-N were measured individually for each goat. For individual VFA molar proportions (acetate, propionate, and butyrate), equal volumes of rumen fluid from six goats per group were pooled, and single measurements were performed using a gas chromatograph (Fuli GC9790Plus, Fuli Instruments, Wenling, China) equipped with a flame ionization detector and a DB-FFAP capillary column (30 m × 0.25 mm i.d. × 0.25 μm film thickness; Agilent Technologies, Santa Clara, CA, USA). Because these pooled samples lacked biological replication, no statistical analysis was performed for individual VFA molar proportions or the acetate-to-propionate ratio; these variables are reported as descriptive data only. The injector and detector temperatures were 250 °C and 280 °C, respectively. The oven temperature was programmed from 60 °C (held for 2 min) to 120 °C at 10 °C/min, then to 220 °C at 20 °C/min (held for 3 min). Nitrogen was used as the carrier gas at a flow rate of 1.0 mL/min with a split ratio of 1:50, and the injection volume was 1 μL. 2-Ethylbutyric acid (Sigma-Aldrich, St. Louis, MO, USA) was added as the internal standard at a final concentration of 1.0 mM. NH_3_-N concentration was measured by the phenol-sodium hypochlorite colorimetric method [14].

### 2.6. Serum Biochemical and Hormonal Assays (Goats)

Serum total protein (TP), albumin (ALB), blood urea nitrogen (BUN), glucose (GLU), triglycerides (TG), and total cholesterol (T-CHO) were measured using an automatic biochemical analyzer (Beckman Coulter AU5800, Brea, CA, USA). Serum concentrations of insulin (INS), triiodothyronine (T3), thyroxine (T4), and growth hormone (GH) were determined using enzyme-linked immunosorbent assay (ELISA) kits (Elabscience, Wuhan, China) following the manufacturer’s instructions.

### 2.7. Serum Antioxidant and Immune Factor ELISA (Rats)

Serum total antioxidant capacity (T-AOC), superoxide dismutase (SOD) activity, glutathione peroxidase (GSH-Px) activity, and malondialdehyde (MDA) content were measured using colorimetric assay kits (Nanjing Jiancheng Bioengineering Institute, Nanjing, China) according to the manufacturer’s instructions. Serum levels of interferon-γ (IFN-γ), tumor necrosis factor-α (TNF-α), interleukin-6 (IL-6), interleukin-10 (IL-10), and interleukin-1β (IL-1β) were determined using rat-specific enzyme-linked immunosorbent assay (ELISA) kits (Nanjing Jiancheng Bioengineering Institute).

### 2.8. Total RNA Extraction and RT-qPCR from Liver

Total RNA was extracted from rat liver tissues using TRIzol reagent (Invitrogen, Carlsbad, CA, USA). RNA concentration and purity (A260/280 ratio > 1.8) were assessed with a NanoDrop 2000 spectrophotometer (Thermo Fisher Scientific, Waltham, MA, USA). One microgram of total RNA was reverse-transcribed into cDNA using PrimeScript™ RT Master Mix (Takara, Shiga, Japan). Quantitative real-time PCR was performed on a LightCycler 480 II (Roche, Basel, Switzerland) using SYBR Premix Ex Taq™ (Takara). The cycling protocol was: 95 °C for 30 s, followed by 40 cycles of 95 °C for 5 s and 60 °C for 30 s, with a melt-curve analysis to verify amplicon specificity. Gapdh served as the internal control, and its stable expression across groups was confirmed by geNorm analysis using qBase+ (version 3.2; Biogazelle, Zwijnaarde, Belgium; M < 0.5). Only one reference gene (Gapdh) was used in this study; the use of at least two reference genes would have been preferable, and this is acknowledged as a limitation. The amplification efficiency for each primer pair was not determined; however, melt-curve analysis confirmed a single amplification product for each gene. Relative gene expression was calculated using the 2^−ΔΔCt^ method without efficiency correction. Gene names are reported according to the official nomenclature (*Ifng*, *Stat1*, *Irf1*, *Tnf*, *Il6*, *Il1b*, *Il10*, *Tlr4*, *Nfe2l2*, *Keap1*, *Hmox1*, *Nqo1*, *Gpx1*, *Sod1*, and *Cat*), and protein names are reported as *IFN-γ*, *STAT1*, *IRF1*, *TNF-α*, *IL-6*, *IL-1β*, *IL-10*, *TLR4*, *NRF2*, *KEAP1*, *HO-1*, *NQO1*, *GPX1*, *SOD1*, and *CAT*. Primers were synthesized by Sangon Biotech (Shanghai, China), and their sequences are provided in Appendix A Table A2.

### 2.9. Liver Protein Extraction and Western Blot

Liver tissues were homogenized in RIPA lysis buffer (Beyotime, Shanghai, China) supplemented with phosphatase inhibitors (PhosSTOP, Roche, Basel, Switzerland) and protease inhibitors (cOmplete, Roche, Basel, Switzerland) to extract total, cytoplasmic, and nuclear proteins using a nuclear extraction kit (Beyotime, Shanghai, China). Protein concentrations were determined using a BCA protein assay kit (Beyotime). Equal amounts of protein (30 μg per lane) were separated by SDS-PAGE and electrotransferred onto PVDF membranes (Millipore, Billerica, MA, USA). After blocking with 5% non-fat milk (Beyotime, Shanghai, China) for 1 h at room temperature, membranes were incubated overnight at 4 °C with primary antibodies against p-STAT1 (Tyr701), total STAT1, TNF-α, IL-6, IL-1β, p-NF-κB p65 (Ser536), total NF-κB p65, Nrf2, Keap1, HO-1, and GPx1 (all from Abcam, Cambridge, UK; diluted according to the manufacturer’s instructions). After washing three times with TBST (Tris-buffered saline with Tween 20; prepared in-house with reagents from Sinopharm Chemical Reagent Co., Ltd., Shanghai, China), membranes were incubated with HRP-conjugated secondary antibodies (Beyotime, diluted 1:2000) for 1 h at room temperature. Signals were visualized using an enhanced chemiluminescence kit (Thermo Fisher Scientific, Waltham, MA, USA) and captured with a ChemiDoc imaging system (Bio-Rad, Hercules, CA, USA). Densitometric quantification was performed using ImageJ software (version 1.53t; National Institutes of Health, Bethesda, MD, USA). β-actin was used as the loading control for total protein. Nuclear Nrf2 was normalized to total Nrf2. *n* = 3 independent biological replicates per treatment were used for Western blot analysis, whereas *n* = 6 was used for qPCR and ELISA. This difference reflects the larger amount of protein required for Western blotting.

### 2.10. 16S rRNA Gene Sequencing and Bioinformatics

Total genomic DNA was extracted from rat fecal samples using a stool DNA extraction kit (Qiagen, Hilden, Germany). The V3-V4 hypervariable regions of the 16S rRNA gene were amplified with primers CCTACGGGNGGCWGCAG and GACTACHVGGGTATCTAATCC, and sequenced on an Illumina MiSeq platform (Illumina, San Diego, CA, USA) in paired-end mode. Raw reads were quality-filtered, assembled, and denoised using the QIIME 2 pipeline (version 2022.2; QIIME 2 Development Team, https://qiime2.org, accessed on 10 August 2026), and amplicon sequence variants (ASVs) were generated via the DADA2 algorithm (version 1.16; R package). Forward and reverse reads were truncated to 220 and 200 bp, respectively, and quality-filtered with maxEE = 2, truncQ = 2, maxN = 0, and minLen = 50 bp. The number of effective reads per sample averaged 75,320 ± 9450, and a total of 2356 ASVs were obtained. Alpha diversity indices (Shannon, Simpson, ACE, and Chao1) and Coverage were calculated after rarefaction to 30,000 reads per sample, as shown in the rarefaction curve (Supplementary Figure S2). Negative and positive controls were not included. The percentage of classified reads was 99.2%. Taxonomic assignment was performed against the SILVA 138 database (SILVA, Bremen, Germany) at the genus level. Beta diversity was assessed by Principal Coordinate Analysis (PCoA) based on Bray–Curtis distances, and inter-group differences were tested by ANOSIM with 999 permutations (R = 0.9983, *p* = 0.001). LEfSe analysis was performed using the LEfSe tool (version 1.0; Huttenhower Lab, Harvard T.H. Chan School of Public Health, Boston, MA, USA) with an LDA threshold > 2.0; multiple comparison correction was not applied. Spearman correlation analysis and heatmap visualization were performed in R software (version 4.0.0; R Foundation for Statistical Computing, Vienna, Austria).

### 2.11. Targeted Quantification of Short-Chain Fatty Acids (SCFAs)

Approximately 100 mg of cecal contents were homogenized with ice-cold acidified ultrapure water containing metaphosphoric acid (final concentration 2.5%, *w*/*v*) and 2-ethylbutyric acid as the internal standard (final concentration 1.0 mM). After centrifugation (12,000× *g*, 10 min, 4 °C), the supernatant was filtered through a 0.22-μm membrane and analyzed using an Agilent 7890A-5975C gas chromatography-mass spectrometry system (Agilent Technologies, Santa Clara, CA, USA). Standard solutions of acetate, propionate, and butyrate, as well as the internal standard, were purchased from Sigma-Aldrich (St. Louis, MO, USA). Concentrations of each of the SCFAs were calculated by the internal standard method and expressed as μmol/g of cecal content.

### 2.12. Statistical Analysis

All data are presented as mean ± standard deviation (SD). For repeated measurements, including daily dry matter intake (DMI), daily milk yield, and weekly milk composition, data were first averaged for each animal over the experimental period. The animal was considered the experimental unit; therefore, each animal contributed a single value per variable, and these animal-level values were used for subsequent one-way ANOVA. This approach avoided pseudoreplication and accounted for within-animal dependence. Normality and homogeneity of variance were assessed using the Shapiro–Wilk test and Levene’s test, respectively. Data meeting these assumptions were analyzed by one-way ANOVA. For comparisons among all groups, Tukey’s HSD post hoc test was used; for comparisons versus the control group, Dunnett’s post hoc test was applied. When the assumptions were not met, the Kruskal–Wallis test was applied, followed by Dunn’s post hoc test for pairwise comparisons. Spearman’s rank correlation coefficient was used to evaluate relationships between gut microbiota, metabolites, and host parameters. Correlation analyses were exploratory; therefore, *p* values were not adjusted for multiple testing. Given the small sample size (*n* = 6), correlation results should be interpreted with caution. A *p* value < 0.05 was considered statistically significant. Linear and quadratic polynomial contrasts were performed across FAP inclusion levels (0%, 5%, 10%, 15%, and 20%) for the principal outcomes, including average daily gain (ADG), dry matter intake (DMI), milk yield, milk fat, milk protein, somatic cell count (SCC), serum total antioxidant capacity (T-AOC), superoxide dismutase (SOD), glutathione peroxidase (GSH-Px), and malondialdehyde (MDA). When the quadratic term was significant (*p* < 0.05), the extremum was estimated from the quadratic regression model as x = −b_1_/(2b_2_). Analyses were performed using SPSS 26.0 (IBM Corp., Armonk, NY, USA) and GraphPad Prism 9.0 (GraphPad Software, San Diego, CA, USA).

## 3. Results

### 3.1. Nutritional Composition and Amino Acid Profile of Raw Apple Pomace

On a dry matter (DM) basis, raw apple pomace contained 4.23% crude protein (CP), 3.61% ether extract (EE), and 2.38% ash; its nutritional composition is summarized in Table 1. It was characterized by high fiber fractions, with neutral detergent fiber (NDF) and acid detergent fiber (ADF) contents of 44.87% and 34.52%, respectively. The non-fiber carbohydrate (NFC) content was 44.91%, resulting in an NDF/CP ratio of 10.61. The calcium (Ca) and phosphorus (P) contents were 0.12% and 0.08%, respectively, giving a Ca/P ratio of 1.50.

**Figure 2:**
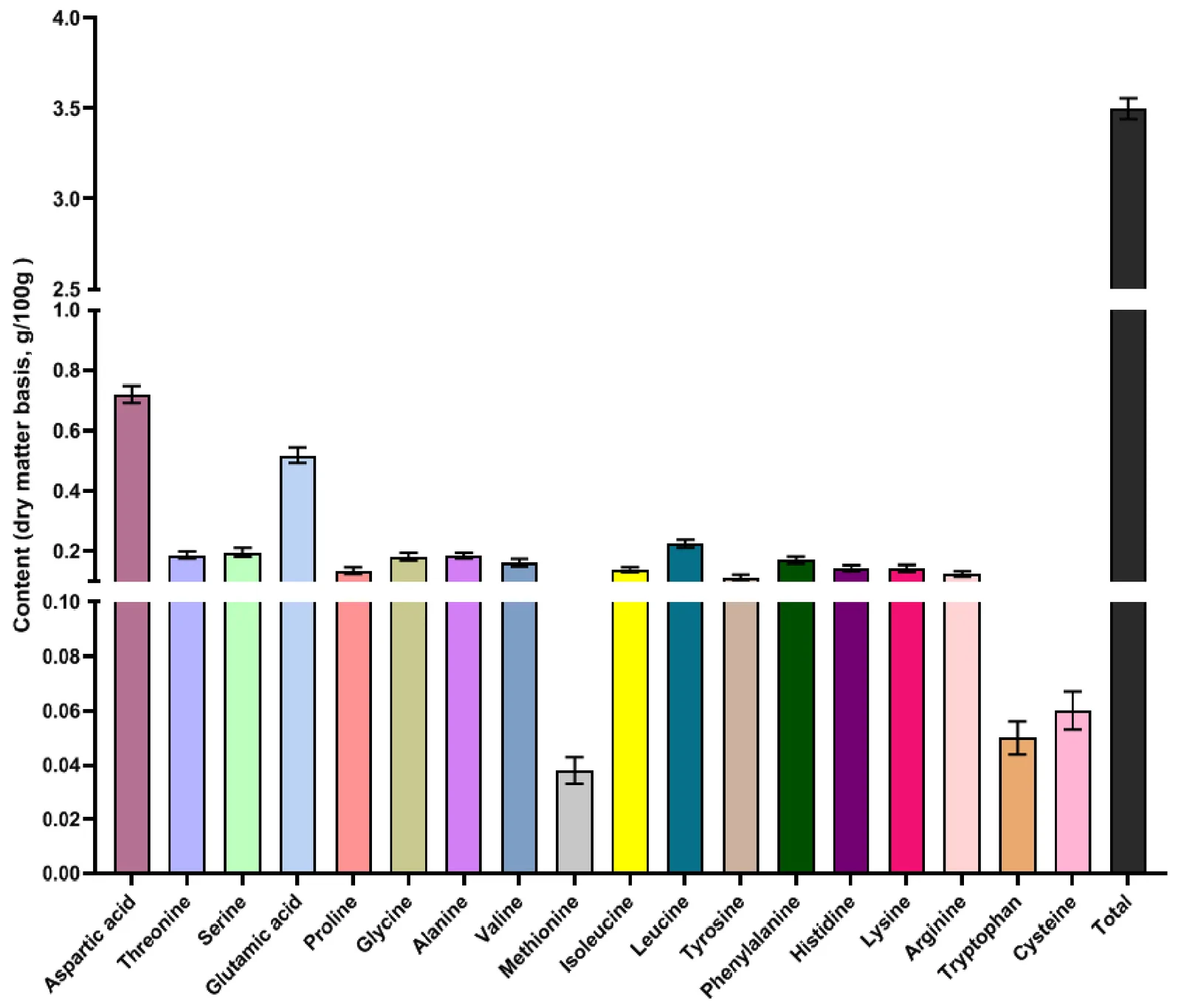
Amino acid profile of raw apple pomace. Different colors are used only to distinguish individual amino acids; the x-axis indicates the name of each amino acid.

The total amino acid content of raw apple pomace was 3.500 g/100 g DM, and its amino acid profile is presented in Figure 2 and Supplementary Table S4. Aspartic acid (0.720 g/100 g) and glutamic acid (0.518 g/100 g) were the most abundant amino acids. Among the essential amino acids, leucine exhibited the highest concentration (0.225 g/100 g), whereas methionine (0.038 g/100 g) and tryptophan (0.050 g/100 g) were the least abundant.

### 3.2. Effects of Fermented Apple Pomace Inclusion Level on Dietary Nutrient Composition and Amino Acid Profile

**Figure 3:**
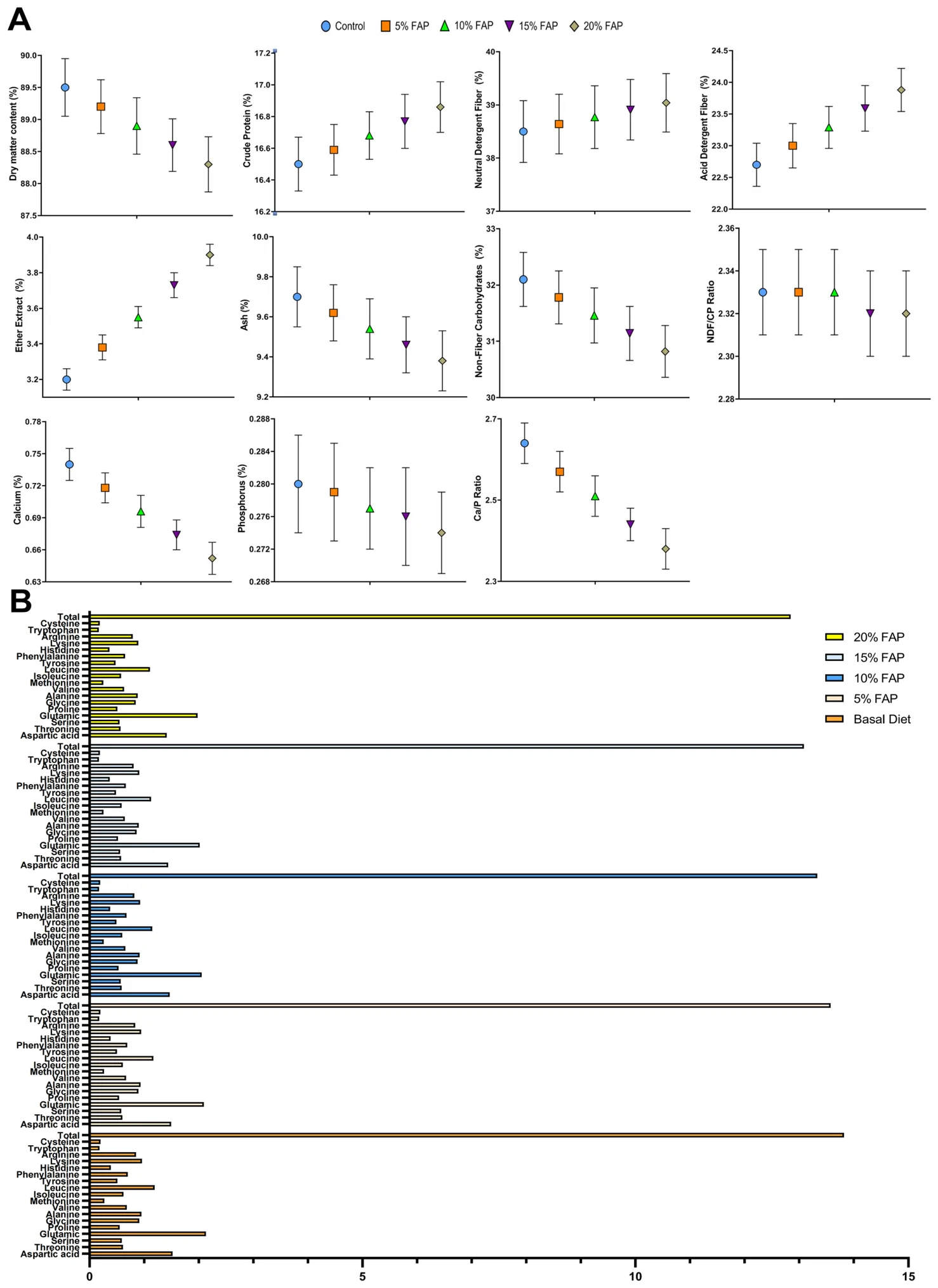
Effects of graded levels of fermented apple pomace (FAP) on the conventional nutrient composition (**A**) and amino acid profile (**B**) of the experimental diets. (**A**) The x-axis represents the dietary inclusion levels of FAP (Control [basal diet], 5%, 10%, 15%, and 20%). The y-axis represents the nutrient contents. Data points are presented as means ± standard deviation (SD) (*n* = 6). (**B**) The horizontal bar chart displays the amino acid contents of the experimental diets. The color-coded bars represent the different dietary treatments: Basal Diet (Control), 5% FAP, 10% FAP, 15% FAP, and 20% FAP. Values are expressed as g/100 g of diet on a dry matter basis. Abbreviations: FAP, fermented apple pomace; DM, dry matter; CP, crude protein; NDF, neutral detergent fiber; ADF, acid detergent fiber; EE, ether extract; NFC, non-fiber carbohydrates; Ca, calcium; P, phosphorus; AA, amino acid.

Dietary dry matter (DM) content decreased linearly from 89.50% to 88.30% as fermented apple pomace (FAP) inclusion increased from 0% to 20% (*p* < 0.001). Compared with the control, crude protein (CP) content increased linearly from 16.50% to 16.86% (*p* < 0.001), accompanied by linear increases in neutral detergent fiber (NDF; 38.50% to 39.04%) and acid detergent fiber (ADF; 22.70% to 23.88%) (*p* < 0.01). Similarly, ether extract (EE) increased linearly from 3.20% to 3.90% (*p* < 0.001). Conversely, ash and non-fiber carbohydrates (NFC) decreased linearly from 9.70% to 9.38% and from 32.10% to 30.82%, respectively (*p* < 0.01). The NDF/CP ratio remained stable across groups (2.33 to 2.32; *p* > 0.05). Calcium content decreased linearly from 0.740% to 0.652% (*p* < 0.001), whereas phosphorus content decreased slightly from 0.280% to 0.274% (*p* = 0.060). Consequently, the Ca/P ratio declined from 2.64 in the control to 2.38 in the 20% FAP group, with the 15% FAP group exhibiting a ratio of 2.44, which fell within the recommended range of 2.0–2.5:1 for ruminants (Figure 3A; Supplementary Table S5).

Regarding the amino acid profile, compared with the control, total amino acid content decreased linearly from 13.820 g/100 g to 12.842 g/100 g in the 20% FAP group (*p* < 0.05; Supplementary Table S6). The total amino acid content in the 15% FAP group was 13.086 g/100 g, representing a significant decrease compared to the control (*p* < 0.05). Reductions in essential amino acids, including lysine, methionine, and leucine, were observed across treatments, with significant linear declines (*p* < 0.001) (Figure 3B; Supplementary Table S6).

### 3.3. Effects of Graded Levels of Fermented Apple Pomace Supplementation on Growth Performance and Milk Quality in Lactating Dairy Goats

**Figure 4:**
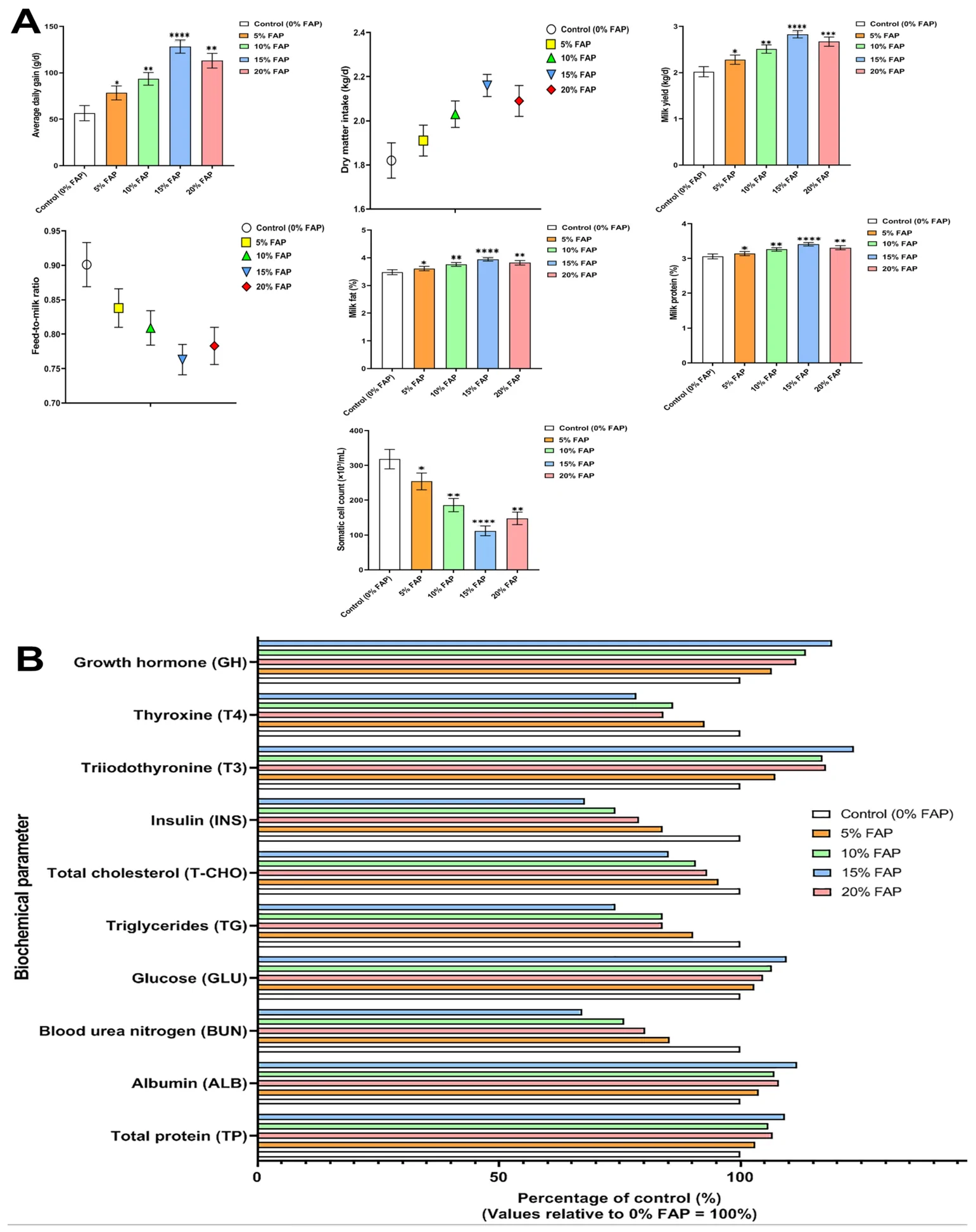
Effects of graded levels of fermented apple pomace (FAP) supplementation on growth performance, milk quality, and serum metabolic, biochemical, and hormonal indices in lactating dairy goats. (**A**) Growth performance (initial body weight, final body weight, average daily gain, dry matter intake, milk yield, and feed-to-milk ratio) and milk quality (milk fat, milk protein, and somatic cell count). (**B**) Serum metabolic, biochemical, and hormonal indices (total protein, albumin, blood urea nitrogen, glucose, triglycerides, total cholesterol, insulin, T3, T4, and growth hormone); values are expressed as a percentage relative to the control (0% FAP) group. Data are presented as mean ± standard deviation (*n* = 6). Data were analyzed using one-way ANOVA followed by Tukey’s HSD post hoc test. Significance markers indicate differences from the control (0% FAP) group: * *p* < 0.05, ** *p* < 0.01, *** *p* < 0.001, **** *p* < 0.0001. The quadratic term was significant for ADG, milk yield, milk fat, milk protein, and SCC (*p* < 0.05), with estimated peak responses at approximately 15% FAP for ADG, milk yield, milk fat, and milk protein, and an estimated nadir at approximately 15% FAP for SCC. Detailed statistical results and polynomial contrasts are provided in Supplementary Tables S7, S8 and S17. Abbreviations: FAP, fermented apple pomace; ADG, average daily gain; SCC, somatic cell count; T3, triiodothyronine; T4, thyroxine.

Data for daily dry matter intake, daily milk yield, and weekly milk composition were averaged per animal over the experimental period before statistical analysis; therefore, *n* = 6 refers to the number of animals per treatment. No significant differences were observed in initial body weight among groups (55.0–55.5 kg, *p* > 0.05), ensuring baseline consistency across experimental treatments (Supplementary Table S7). All productive performance parameters, including dry matter intake (DMI), milk yield, and feed conversion ratio, showed distinct curvilinear responses across the graded FAP inclusion levels (Figure 4A). Specifically, increasing FAP inclusion was associated with increased DMI, which rose from 1.82 kg/d in the control to 2.16 kg/d in the 15% FAP group (an 18.7% increase, *p* < 0.001), before slightly declining to 2.09 kg/d in the 20% group (*p* < 0.01). Milk yield showed a similar inverted U-shaped pattern, being higher in the 15% FAP group (2.83 kg/d) than in the control (2.02 kg/d; a 40.1% increase, *p* < 0.0001), and then lower in the 20% group (2.67 kg/d; *p* < 0.001). The feed-to-milk ratio showed the opposite trend, decreasing from 0.901 in the control to 0.763 in the 15% FAP group (a 15.3% reduction, *p* < 0.001), and rising to 0.783 in the 20% group (*p* < 0.01). Concurrently, the 15% FAP group had the greatest final body weight (61.2 kg, *p* < 0.05) and an average daily gain (ADG) of 128.3 g/d, which was 127.1% higher than that of the control (56.5 g/d, *p* < 0.0001); the 20% group had an ADG of 113.0 g/d (*p* < 0.01). The magnitude of improvement in ADG closely paralleled that of milk yield, consistent with an improvement in body weight gain under the conditions of the present study.

Regarding milk quality, the 15% FAP group had the highest milk fat and milk protein contents among all treatments, at 3.94% and 3.41%, respectively, corresponding to 13.2% and 11.4% improvements over the control (3.48% and 3.06%, respectively; *p* < 0.0001); the 20% group also had significantly higher milk fat (3.82%) and milk protein (3.31%) than the control (*p* < 0.01). Somatic cell count (SCC), a critical indicator of udder health and raw milk quality, was lower with increasing FAP inclusion, decreasing from 318 × 10^3^/mL in the control to 112 × 10^3^/mL in the 15% FAP group, a reduction of 64.8% (*p* < 0.0001), and was 148 × 10^3^/mL in the 20% group (*p* < 0.01). The reduction in SCC is consistent with an improvement in udder health; however, the underlying inflammatory or microbiological mechanisms were not directly assessed.

Notably, most measured parameters exhibited a consistent attenuation at the 20% FAP inclusion level. The evaluated indices showed a highly congruent inverted U-shaped dose–response relationship. This pattern supports 15% FAP as the inclusion level associated with the most favorable responses among the tested levels for dietary supplementation in dairy goats (Figure 4A; Supplementary Tables S7 and S17).

### 3.4. Regulatory Effects of Fermented Apple Pomace on Serum Metabolic Profiles and Endocrine Indices in Dairy Goats

Dietary FAP supplementation exerted significant regulatory effects on the serum metabolic profile and endocrine indices of dairy goats (Figure 4B; Supplementary Table S8). The changes in serum biochemical indices were observed alongside changes in rumen fermentation; however, because individual VFA data were obtained from pooled samples, these relationships should be interpreted as associative rather than causal. The 15% FAP group exhibited maximal elevations in total protein, albumin, and glucose, which increased from 68.2 g/L, 32.5 g/L, and 3.42 mmol/L in the control to 74.5 g/L, 36.3 g/L, and 3.75 mmol/L, respectively, representing increases of 9.2%, 11.7%, and 9.6% (*p* < 0.01). In contrast, serum urea nitrogen (BUN) decreased from 5.8 mmol/L in the control to 3.90 mmol/L in the 15% FAP group, a reduction of 32.8% (*p* < 0.0001), which closely paralleled the decline in ruminal NH_3_-N. These changes are consistent with altered nitrogen metabolism; however, because nitrogen balance was not measured, they should not be interpreted as direct evidence of improved nitrogen retention. The endocrinological profile further elucidated the underlying mechanisms of metabolic modulation: growth hormone (GH) and T3 levels peaked in the 15% FAP group, increasing from 3.52 ng/mL and 1.24 ng/mL to 4.19 ng/mL and 1.53 ng/mL, respectively, representing increases of 19.0% (*p* < 0.01) and 23.4% (*p* < 0.001), whereas insulin decreased from 12.4 μIU/mL to 8.4 μIU/mL, a reduction of 32.3% (*p* < 0.001). Meanwhile, thyroxine (T4) decreased from 48.6 ng/mL to 38.1 ng/mL, a reduction of 21.6% (*p* < 0.01). These changes were accompanied by reductions in triglycerides and total cholesterol from 0.31 mmol/L and 2.16 mmol/L to 0.23 mmol/L and 1.84 mmol/L, respectively, representing decreases of 25.8% and 14.8% (*p* < 0.01), collectively indicating a systemic metabolic state characterized by attenuated lipolysis, enhanced protein synthesis, and altered glucose–insulin homeostasis. However, because insulin sensitivity was not directly measured, these findings should be interpreted as changes in circulating insulin and glucose concentrations rather than as direct evidence of improved insulin sensitivity. The endocrine combination of low insulin, elevated GH, and elevated T3 typically directs nutrient partitioning toward anabolism, providing an endocrinological basis for the synergistic improvement of growth and lactational performance in dairy goats. Notably, all of the aforementioned serum metabolic and endocrine parameters exhibited clear reversal trends at the 20% FAP inclusion level. Collectively, these findings indicate that 15% FAP supplementation was associated with changes in rumen fermentation and nitrogen metabolism. These changes were accompanied by a serum endocrinological profile characterized by low insulin, elevated GH, and elevated T3. This endocrine pattern is consistent with an anabolic state that may support growth and lactation in dairy goats. However, because individual VFA data were obtained from pooled samples, the proposed role of propionate should be regarded as a descriptive trend rather than a confirmed mechanism.

### 3.5. Regulatory Effects of Fermented Apple Pomace on Rumen Fermentation Patterns in Dairy Goats

**Figure 5:**
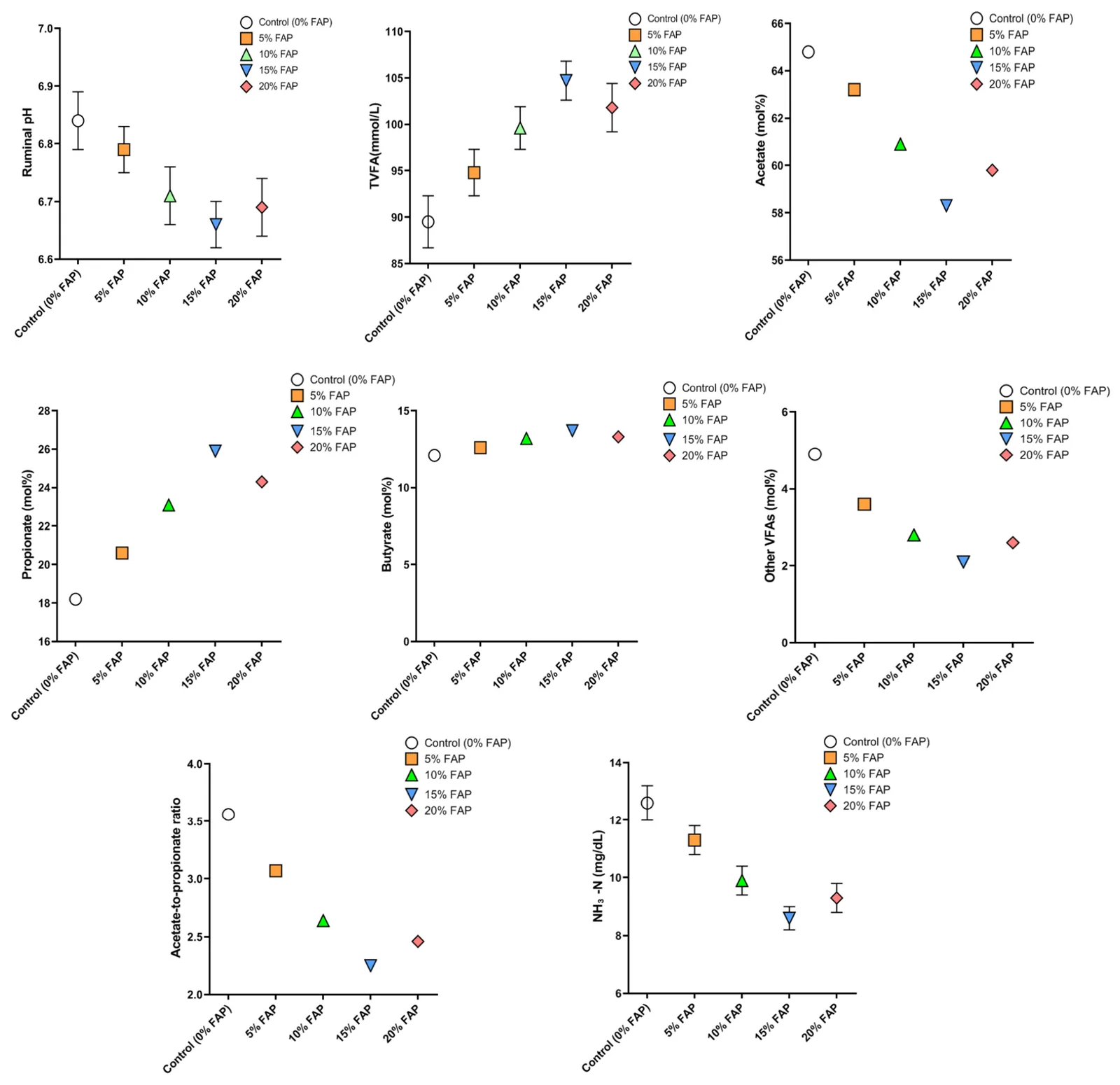
Effects of graded levels of fermented apple pomace (FAP) supplementation on rumen fermentation parameters in lactating dairy goats. Rumen fermentation parameters (pH, TVFA, NH_3_-N, acetate, propionate, butyrate, and acetate-to-propionate ratio). For pH, TVFA, and NH_3_-N, data are presented as mean ± SD (*n* = 6 per treatment) and were analyzed using one-way ANOVA followed by Tukey’s HSD post hoc test. For the acetate, propionate, butyrate, and acetate-to-propionate ratio, values were obtained from pooled rumen fluid samples (equal volumes from six goats per group) and are therefore single measurements without biological replication. No statistical analysis was performed for these variables, and no significance markers are shown. These pooled VFA proportions should be interpreted as descriptive trends only. Detailed statistical results and polynomial contrasts are provided in Supplementary Table S9. Abbreviations: FAP, fermented apple pomace; TVFA, total volatile fatty acids; NH_3_-N, ammonia nitrogen.

Dietary FAP supplementation was associated with changes in the rumen fermentation pattern in dairy goats (Figure 5; Supplementary Table S9). Ruminal pH remained relatively stable across treatments, declining only slightly from 6.84 in the control to 6.66 in the 15% FAP group and 6.69 in the 20% group, all within the normal physiological range for ruminants (6.5–7.0). TVFA concentration increased from 89.5 mmol/L in the control to 104.7 mmol/L in the 15% FAP group, before declining to 101.8 mmol/L in the 20% group. Ruminal NH_3_-N concentration decreased from 12.6 mg/dL in the control to 8.6 mg/dL in the 15% FAP group, consistent with lower ruminal NH_3_-N concentration. However, nitrogen balance and retention were not directly measured.

In pooled rumen fluid, the molar proportion of propionate appeared to increase from 18.2% in the control to 25.9% in the 15% FAP group, accompanied by an apparent decline in acetate from 64.8% to 58.3%, resulting in an apparent reduction in the acetate-to-propionate ratio from 3.56 to 2.25. The pooled VFA analysis showed an apparent increase in propionate proportion at 15% FAP; however, because samples were pooled within treatment, these values should be regarded as descriptive observations and not as treatment effects. Butyrate proportion appeared modestly elevated from 12.1% to 13.7%. However, because these individual VFA and ratio data were obtained from pooled samples (equal volumes from six goats per group) and were single measurements without statistical replication, no statistical inference is warranted. These apparent shifts should therefore be interpreted as descriptive trends only. The statistically supported fermentation changes—elevated TVFA and reduced NH_3_-N—suggest enhanced rumen fermentation and altered nitrogen metabolism. However, nitrogen balance and retention were not directly measured. Because these pooled VFA data are descriptive only, the apparent propionate-type shift should not be regarded as a confirmed mechanism and should not be used to explain subsequent metabolic or production responses. Any potential contribution of propionate to gluconeogenic precursor supply remains hypothetical and requires confirmation with individual-animal VFA measurements.

At the 20% FAP inclusion level, TVFA and NH_3_-N were intermediate between the control and 15% FAP groups, consistent with 15% being associated with the most favorable responses among the tested levels. However, individual VFA responses at 20% cannot be statistically evaluated because of the pooled-sample design.

### 3.6. Dose-Dependent Effects of Fermented Apple Pomace on Serum Antioxidant Parameters in Rats

**Figure 6:**
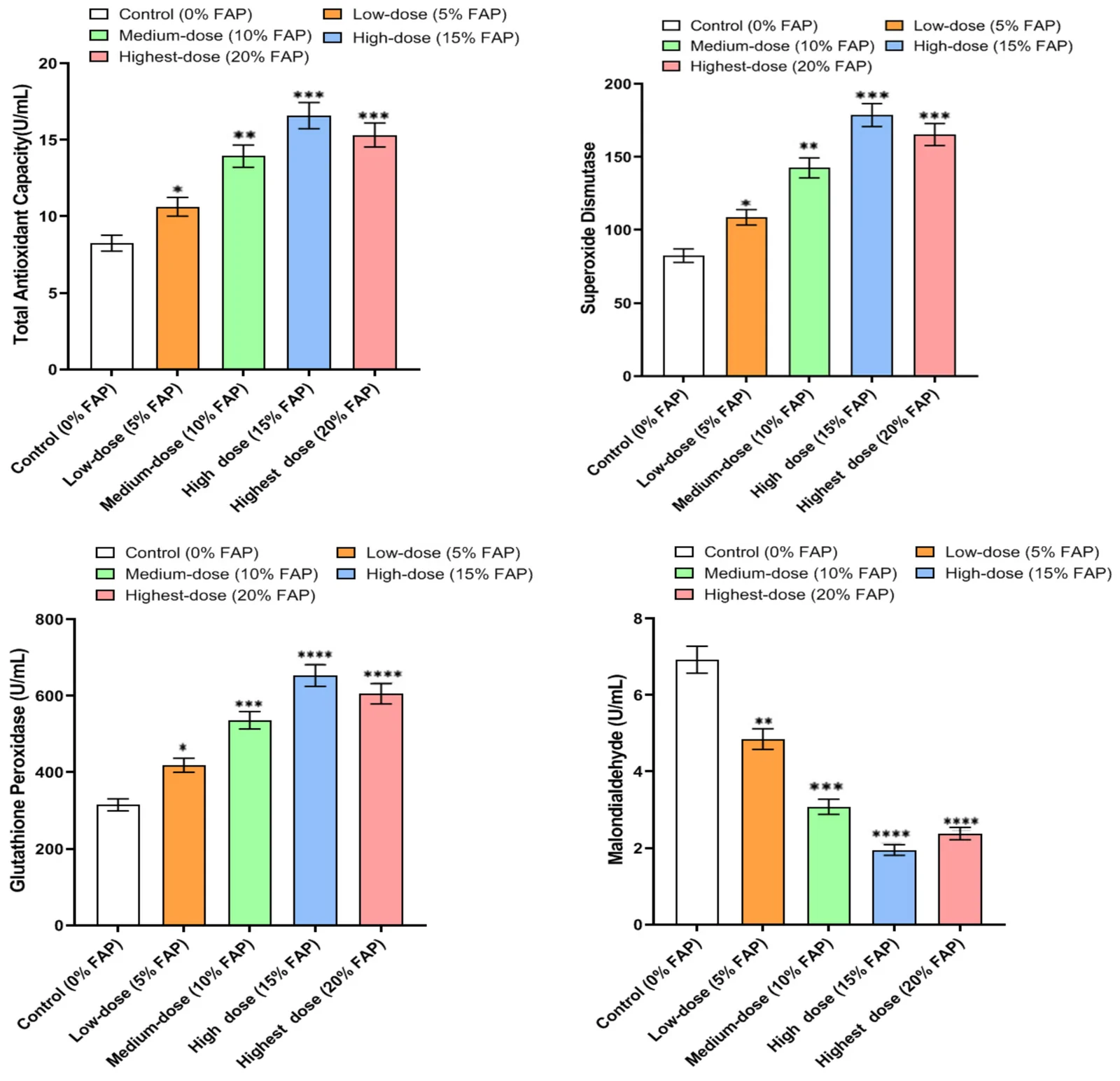
Effects of fermented apple pomace (FAP) supplementation on serum antioxidant parameters in rats. Values are presented as mean ± standard deviation (*n* = 10 per group). * *p* < 0.05, ** *p* < 0.01, *** *p* < 0.001, **** *p* < 0.0001 vs. the control group (0% FAP) (one-way ANOVA followed by Dunnett’s post hoc test). T-AOC, total antioxidant capacity; SOD, superoxide dismutase; GSH-Px, glutathione peroxidase; MDA, malondialdehyde. Quadratic dose–response analysis was significant for T-AOC, SOD, GSH-Px, and MDA (quadratic *p* < 0.05), with estimated peak responses at approximately 15% FAP for T-AOC, SOD, and GSH-Px, and an estimated nadir at approximately 15% FAP for MDA.

To preliminarily investigate the mechanistic basis of the antioxidant potential of FAP, a rat model was employed in this study. With increasing FAP supplementation from 0% to 20%, the serum antioxidant profile of rats was significantly modulated (Figure 6; Supplementary Table S10). Serum total antioxidant capacity (T-AOC) exhibited a distinct curvilinear response, rising progressively from 8.25 U/mL in the control group to 10.63, 13.94, and peaking at 16.58 U/mL in the 15% FAP group—a 101.0% increase over the control (*p* < 0.001)—followed by a decline to 15.32 U/mL at the 20% inclusion level. Superoxide dismutase (SOD) and glutathione peroxidase (GSH-Px) displayed analogous trends, both peaking in the 15% FAP group at 178.6 and 652.7 U/mL, respectively, representing 116.7% (*p* < 0.001) and 107.1% (*p* < 0.0001) increases over the control, before decreasing to 165.3 and 605.1 U/mL in the 20% group. In contrast, malondialdehyde (MDA) exhibited an inverse pattern, declining from 6.92 nmol/mL in the control to its nadir of 1.95 nmol/mL in the 15% FAP group (a 71.8% reduction, *p* < 0.0001), followed by a rebound to 2.38 nmol/mL at 20% inclusion.

Notably, the highest levels of T-AOC, SOD, and GSH-Px, together with the lowest MDA level, all converged in the 15% FAP group. Across the 5% to 15% range, all parameters displayed stepwise improvements (*p* < 0.05 vs. the control group). The coordinated upregulation of the enzymatic antioxidant defense system (T-AOC, SOD, and GSH-Px) reflects a systemic enhancement of endogenous antioxidant capacity, whereas the marked reduction in MDA provides direct evidence of alleviated oxidative stress in this rodent model. Collectively, the 15% FAP group consistently exhibited the most favorable antioxidant profile, while the 20% group showed a uniform reversal across all indices. The quadratic term was significant for T-AOC (*p* = 0.003), SOD (*p* = 0.007), GSH-Px (*p* = 0.021), and MDA (*p* = 0.004), with estimated peak (or nadir) responses at approximately 15% FAP (Supplementary Table S17). These findings in rats provide a preliminary mechanistic reference for the antioxidant potential of FAP, though further validation in dairy goats is warranted to confirm whether similar regulatory effects occur in ruminants.

### 3.7. Regulatory Mechanisms of Fermented Apple Pomace on Inflammatory Signaling and the Nrf2 Antioxidant Pathway

**Figure 7:**
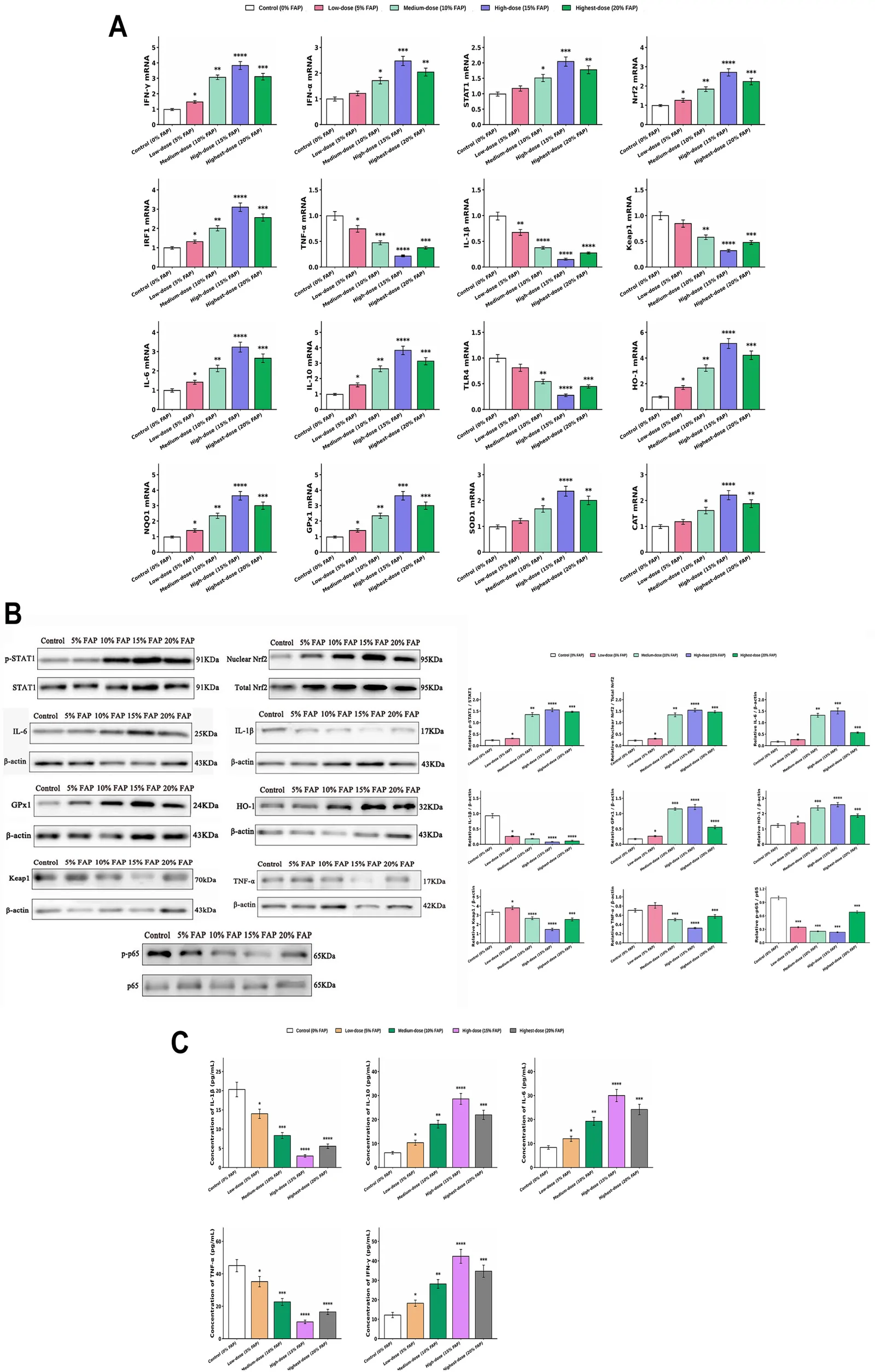
Multilevel (transcription–translation–secretion) integrative regulation of interferon signaling, classical inflammatory, and Nrf2 antioxidant pathways by fermented apple pomace (FAP) and dose-dependent validation. (**A**) qPCR analysis of gene expression. (**B**) Western blot bands and densitometric quantification. (**C**) ELISA analysis of serum cytokine levels. Values are presented as mean ± standard deviation (*n* = 6 for qPCR and ELISA; *n* = 3 for Western blot). * *p* < 0.05, ** *p* < 0.01, *** *p* < 0.001, **** *p* < 0.0001 vs. the control group (0% FAP) (one-way ANOVA followed by Dunnett’s post hoc test).

To elucidate the molecular mechanisms underlying the immunomodulatory and antioxidant effects of FAP, we systematically evaluated the dose-dependent regulation of interferon signaling, classical inflammatory pathways, and the Nrf2 antioxidant pathway at three levels—gene transcription, protein translation (including phosphorylation modifications), and functional secretion—using RT-qPCR, Western blot, and ELISA in rats (Figure 7).

At the level of interferon signaling (Figure 7A,B), FAP supplementation was associated with enhanced immune defense response in rats. With increasing FAP inclusion from 0% to 15%, the mRNA expression of IFN-γ and its downstream signal transducer STAT1, as well as the effector transcription factor IRF1, were upregulated by 3.85-, 2.05-, and 3.12-fold, respectively (Figure 7A; all *p* < 0.0001). Concurrently, the phosphorylation ratio of STAT1 (p-STAT1/STAT1) increased by 3.45-fold, synchronously peaking in the 15% group (Figure 7B; *p* < 0.0001). These indices exhibited significant attenuation at the 20% inclusion level.

At the level of classical inflammatory signaling (Figure 7A–C), FAP supplementation was associated with suppression of the TLR4/NF-κB signaling axis and its downstream pro-inflammatory cascade in rats. In the 15% group, TLR4 mRNA expression decreased to 0.28-fold of the control (Figure 7A; *p* < 0.0001), and phosphorylation of the core transcription factor NF-κB p65 (p-p65/p65) was drastically suppressed to 0.12-fold (Figure 7B; *p* < 0.0001). The downstream pro-inflammatory cytokines TNF-α and IL-1β were simultaneously reduced to their lowest levels across all groups at the transcriptional (qPCR), intracellular synthesis (Western blot), and secretory (ELISA) levels (Figure 7A–C; all *p* < 0.0001). Conversely, the anti-inflammatory cytokine IL-10 reached its peak mRNA level in the 15% group (a 3.85-fold increase over the control, *p* < 0.0001), establishing a robust anti-inflammatory microenvironment.

At the level of the Nrf2 antioxidant signaling pathway (Figure 7A,B), FAP supplementation was associated with alleviation of the constraint of the negative regulator Keap1 and activation of nuclear translocation of the core transcription factor and its downstream defense systems in rats. In the 15% group, Keap1 mRNA expression was suppressed to 0.32-fold of the control (Figure 7A; *p* < 0.0001), while Western blot analysis revealed a visibly weaker Keap1 protein band. This drove the nuclear Nrf2/total Nrf2 ratio (reflecting Nrf2 nuclear translocation and functional activation) to its highest level across all groups (a 4.35-fold increase over the control, Figure 7B; *p* < 0.0001). Multiple downstream antioxidant target genes (HO-1, NQO1, GPx1, SOD1, and CAT) exhibited synchronized upregulation at the transcriptional level in the 15% group, with HO-1 mRNA reaching 5.15-fold of the control (Figure 7A; *p* < 0.0001). Western blot results further confirmed that the protein translation trends of HO-1 and GPx1 were highly consistent with their mRNA trends, both peaking in the 15% group (Figure 7B), confirming that FAP exerts its antioxidant effects coordinately at both the transcriptional and translational levels in this rodent model.

Notably, IL-6 exhibited a distinct regulatory pattern (Figure 7A–C). Despite the strong suppression of upstream NF-κB phosphorylation and classical pro-inflammatory cytokines (TNF-α and IL-1β) by FAP, IL-6 mRNA, intracellular protein, and serum secretion levels (30.2 pg/mL vs. 8.5 pg/mL in the control, Figure 7C; *p* < 0.0001) were all significantly upregulated in the 15% group. Together with the concurrently elevated IL-10, this observation suggests that the surge in IL-6 under 15% FAP treatment may not be mediated through the classical NF-κB-dependent pro-inflammatory pathway, but rather may involve STAT3-mediated tissue repair and immune remodeling, reflecting the finely tuned bidirectional immunomodulatory function of FAP. However, this speculation requires validation through phospho-STAT3 detection and hepatic proliferation markers.

In summary, in rats, 15% FAP was associated with coordinated regulation of IFN-mediated immune defense activation, classical NF-κB inflammatory suppression, and Nrf2-mediated antioxidant defense enhancement at the transcriptional, translational, and secretory levels. The consistent attenuation of all measured targets at the 20% inclusion level further supports 15% FAP as the inclusion level associated with the most favorable responses among the tested levels in this rodent model. However, these mechanistic findings should not be directly extrapolated to dairy goats, because hepatic Nrf2/NF-κB signaling and cecal SCFAs/microbiota were not measured in goats.

### 3.8. Effects of Fermented Apple Pomace on Fecal Microbiota Remodeling and Host–Microbe Associations

**Figure 8:**
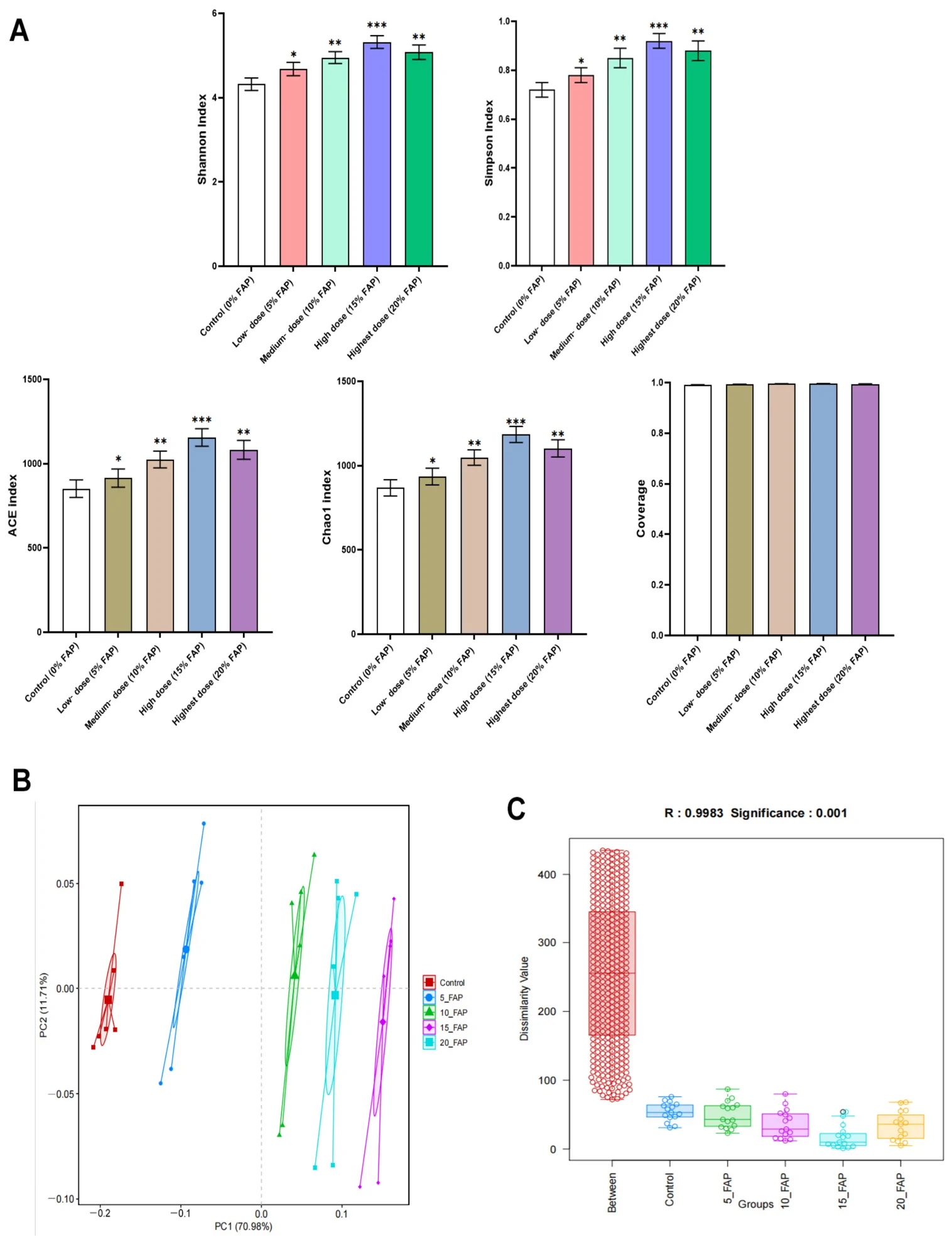
Effects of fermented apple pomace (FAP) supplementation on alpha and beta diversity of fecal microbiota in rats. (**A**) Alpha diversity indices (Shannon, Simpson, ACE, Chao1, and Coverage). (**B**) Principal coordinate analysis (PCoA) plot based on Bray–Curtis distances showing overall microbial community structure separation among groups. (**C**) ANOSIM boxplot confirming significant inter-group differences. For alpha diversity indices in (**A**), values are presented as mean ± standard deviation (*n* = 6 per group). * *p* < 0.05, ** *p* < 0.01, *** *p* < 0.001 vs. the control group (one-way ANOVA followed by Dunnett’s post hoc test). For panels (**B**,**C**), statistical analyses were performed using ANOSIM based on relative abundance data. FAP, fermented apple pomace; PCoA, principal coordinate analysis; ANOSIM, analysis of similarities.

**Figure 9:**
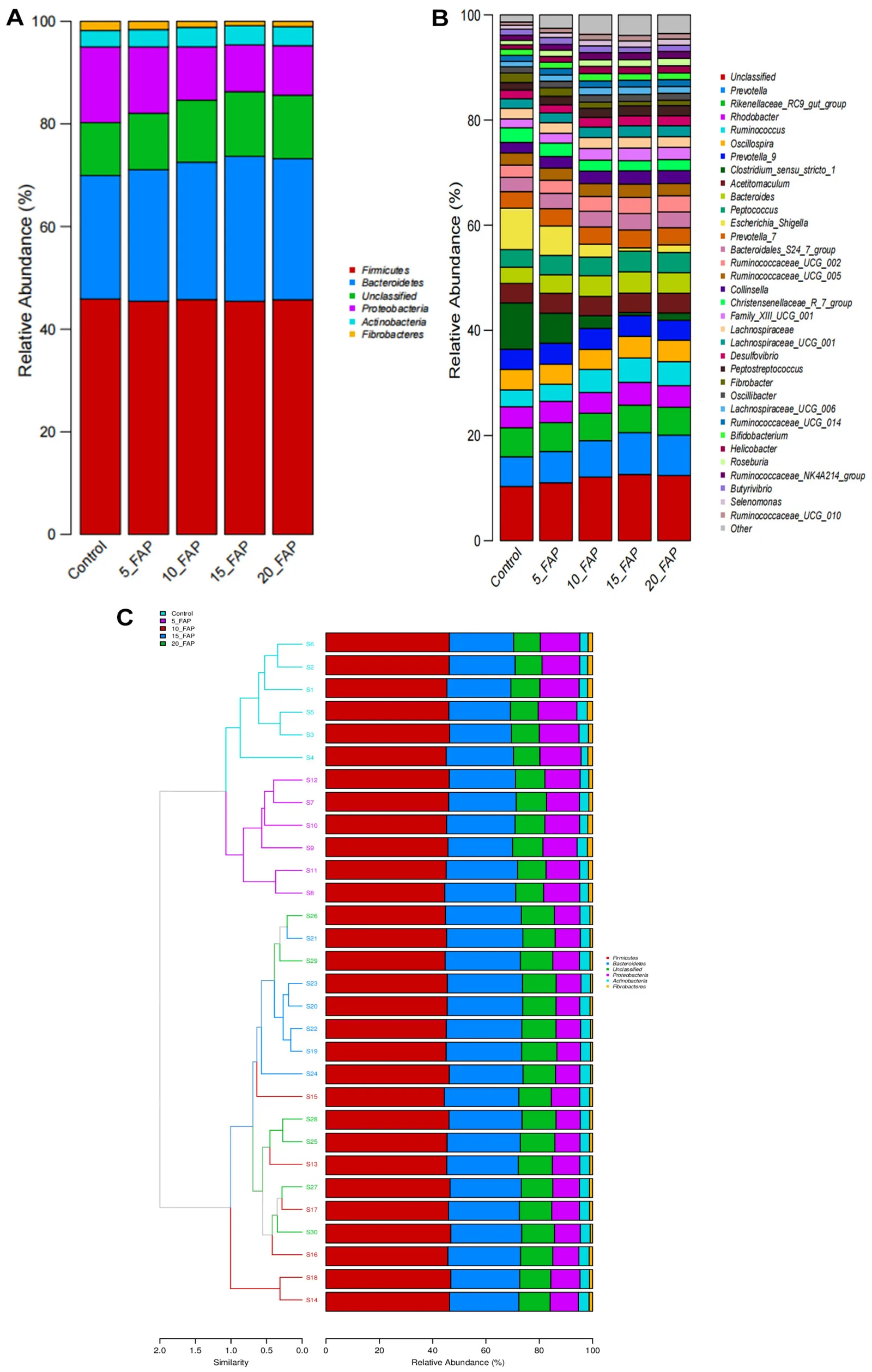
Effects of fermented apple pomace (FAP) supplementation on fecal microbial composition in rats. (**A**) Relative abundance at the phylum level. (**B**) Relative abundance at the genus level. (**C**) Hierarchical clustering tree combined with the genus-level bar plot. Data are presented as relative abundance (%). FAP, fermented apple pomace.

**Figure 10:**
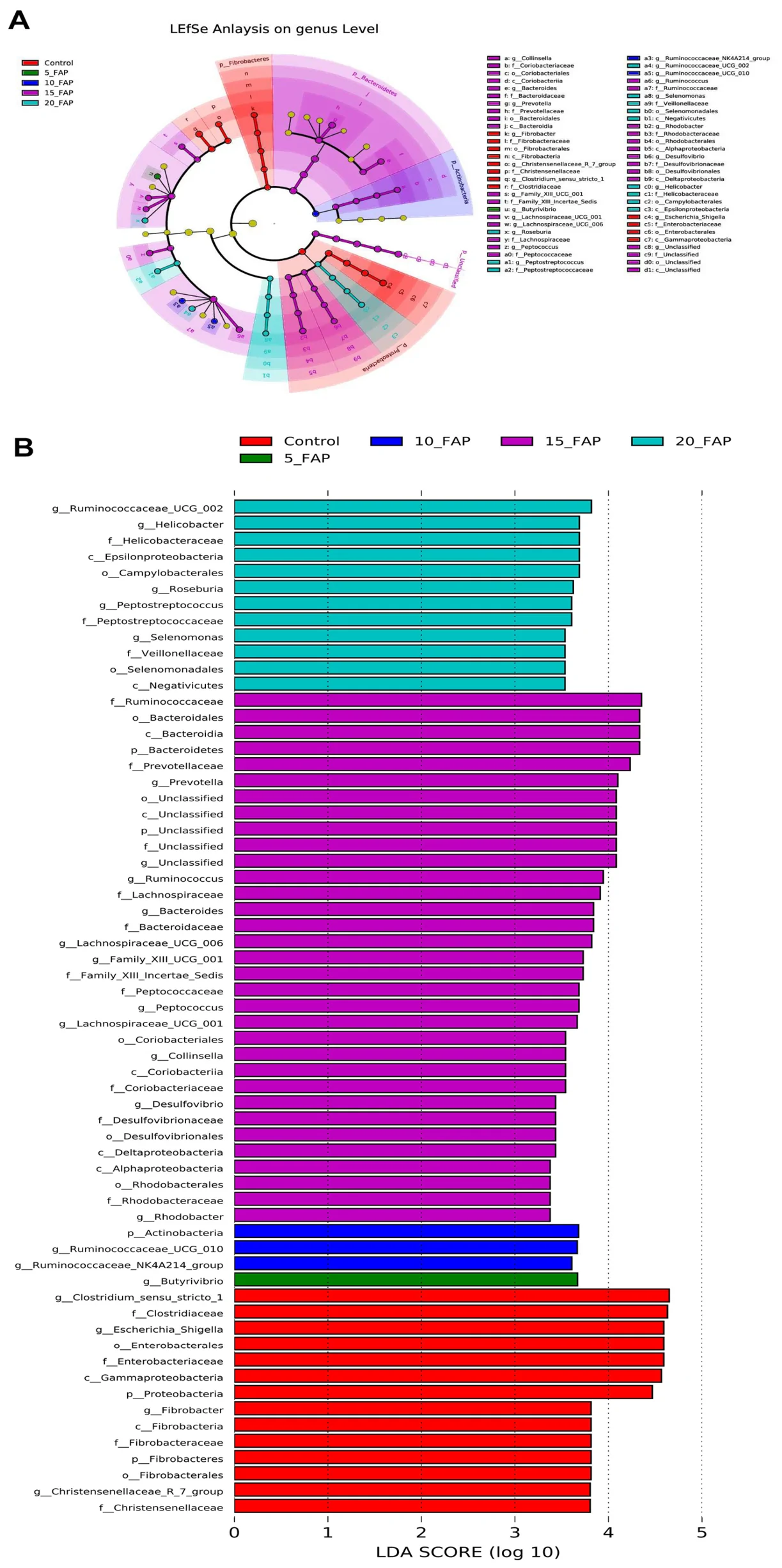
Differential taxa identified by LEfSe in fecal microbiota of rats supplemented with fermented apple pomace (FAP). (**A**) LEfSe cladogram identifying key discriminatory taxa (biomarkers) enriched in each group. (**B**) LDA score bar plot of differentially abundant genera (LDA > 2.0). Statistical analyses were performed using LEfSe (LDA > 2.0) based on relative abundance data. FAP, fermented apple pomace; LDA, linear discriminant analysis.

To investigate whether the gut microbiome serves as an upstream driver of FAP-mediated immunomodulatory and antioxidant effects, high-throughput 16S rRNA sequencing of fecal microbiota was performed in rats (Figure 8, Figure 9 and Figure 10). Alpha diversity analysis of fecal microbiota (Figure 8A; Supplementary Table S12) revealed that the Shannon and Simpson indices exhibited an inverted U-shaped trend with increasing FAP inclusion, both peaking in the 15% FAP group (*p* < 0.001), while the ACE and Chao1 indices followed the same pattern (*p* < 0.001), indicating that 15% FAP supplementation effectively enhanced both species richness and evenness of the gut microbiota; the Coverage index was approximately 1.00 across all groups, confirming that the sequencing depth was sufficient to capture the vast majority of the microbial community. Principal coordinate analysis based on Bray–Curtis distances (Figure 8B) displayed a clear gradient separation among the five groups (PC1 = 70.88%, PC2 = 17.76%), with the 15% and 20% FAP groups located farthest from the control, and ANOSIM analysis (Figure 8C) further confirmed extremely significant inter-group differences (R = 0.9983, *p* = 0.001).

At the phylum level (Figure 9A), Firmicutes and Bacteroidetes were the dominant phyla in all groups, and the Firmicutes/Bacteroidetes (F/B) ratio decreased progressively with increasing FAP inclusion, from 1.98 in the control to 1.52 in the 15% FAP group and 1.60 in the 20% FAP group. At the genus level (Figure 9B), the 15% FAP group was significantly enriched in SCFA-producing beneficial genera, including *Prevotella*, *Ruminococcus*, *Butyrivibrio*, and *Roseburia*, whereas potentially opportunistic pathogens such as Escherichia/Shigella were significantly suppressed.

The sample clustering dendrogram (Figure 9C) showed that the 15% and 20% FAP groups clustered closely on the same branch, and LEfSe analysis (LDA > 2.0) further identified the characteristic signature genera of the 15% FAP group as *Prevotella* (LDA = 4.82), *Ruminococcus* (LDA = 4.16), and *Butyrivibrio* (LDA = 3.95) (Figure 10A,B). This microbial remodeling provides an upstream microbiological basis for the subsequent enhancement of SCFA production and activation of the Nrf2 antioxidant pathway.

### 3.9. Association Analyses Between Gut Microbiota Composition and Host Serum Parameters

**Figure 11:**
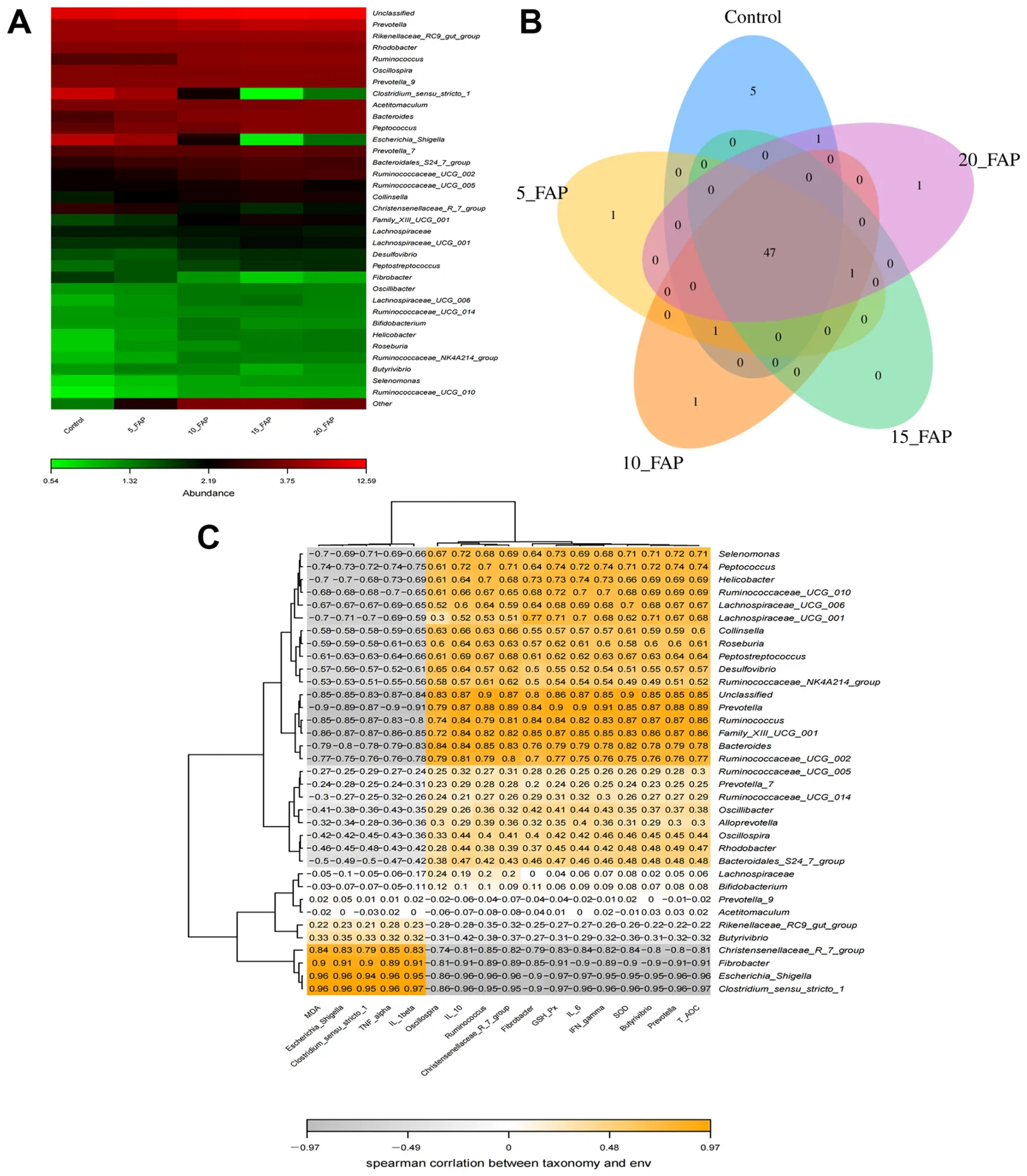
Association analyses between gut microbiota composition and host serum parameters in rats. (**A**) Hierarchical clustering heatmap of the relative abundance of dominant genera across groups. Color intensity from green (low abundance) to red (high abundance) represents relative abundance values. Dendrograms on the left and top show the hierarchical clustering of genera and samples, respectively. (**B**) Venn diagram of shared and unique OTUs among the five groups. The central overlapping region (47) indicates the number of core OTUs shared by all five groups, and each petal indicates the number of unique OTUs specific to the corresponding group. (**C**) Spearman correlation heatmap between dominant genera and host serum parameters (T-AOC, SOD, GSH-Px, MDA, IFN-γ, TNF-α, IL-6, IL-1β, IL-10). Red indicates positive correlation, and blue indicates negative correlation. No significance markers are shown because no correlation reached the 0.05 significance level (for *n* = 6, |r| ≥ 0.811 corresponds to *p* < 0.05). These correlation analyses were exploratory and should be interpreted with caution. T-AOC, total antioxidant capacity; SOD, superoxide dismutase; GSH-Px, glutathione peroxidase; MDA, malondialdehyde; OTU, operational taxonomic unit.

To further explore the functional coupling between the gut microbiome and host systemic status, we performed multilevel association analyses (Figure 11). Hierarchical clustering heatmap analysis (Figure 11A) revealed a clear structural separation of the dominant genera among the different treatment groups. The 15% FAP group exhibited a distinct microbial signature, characterized by a marked enrichment of beneficial genera (e.g., *Prevotella*, *Ruminococcus*, and *Butyrivibrio*), which clearly distinguished it from the control and 20% FAP groups.

Venn diagram analysis (Figure 11B) revealed that the five groups shared 47 core OTUs, accounting for the vast majority of the total OTU sequences, indicating that these shared OTUs constituted the core skeleton of the gut microbiota. The number of unique OTUs in each group was extremely low (5 in the control and only 0–1 in the remaining groups). This suggests that the effect of FAP on the gut microbiome does not depend on introducing a large number of novel exogenous species, but rather on precisely modulating the abundance structure of the shared core probiotics to achieve microbial ecological remodeling.

Microbiota–host correlation analysis (Figure 11C) further characterized these associations. Spearman correlation heatmaps revealed that *Prevotella* and *Butyrivibrio* showed positive numerical correlations with serum T-AOC, SOD, and GSH-Px (r = 0.58–0.75), whereas *Ruminococcus* showed weaker correlations with these parameters. *Prevotella* and *Butyrivibrio* also showed negative numerical correlations with MDA and the pro-inflammatory cytokines TNF-α and IL-1β, while the suppressed genus Escherichia/Shigella exhibited the opposite correlation pattern. However, with *n* = 6, none of these correlations reached statistical significance (|r| ≥ 0.811 required for *p* < 0.05). These association analyses should therefore be considered exploratory and interpreted with caution.

In summary, 15% FAP structurally enriched SCFA-producing beneficial genera while suppressing opportunistic pathogens, thereby remodeling the abundance of core gut microbiota. This microbial shift formed a tight functional coupling with systemic antioxidant and anti-inflammatory capacities.

### 3.10. Fermented Apple Pomace Modulates Gut Microbial Metabolites and Their Associations with Host Antioxidant and Immune Status

To verify whether FAP-induced gut microbiota remodeling translates into functional metabolic signals that activate the downstream Nrf2 antioxidant pathway, we performed targeted quantification of the major short-chain fatty acids (SCFAs)—acetic acid, propionic acid, and butyric acid—in rat cecal contents, and analyzed their correlations with microbial abundance and host antioxidant/immune parameters.

**Figure 12:**
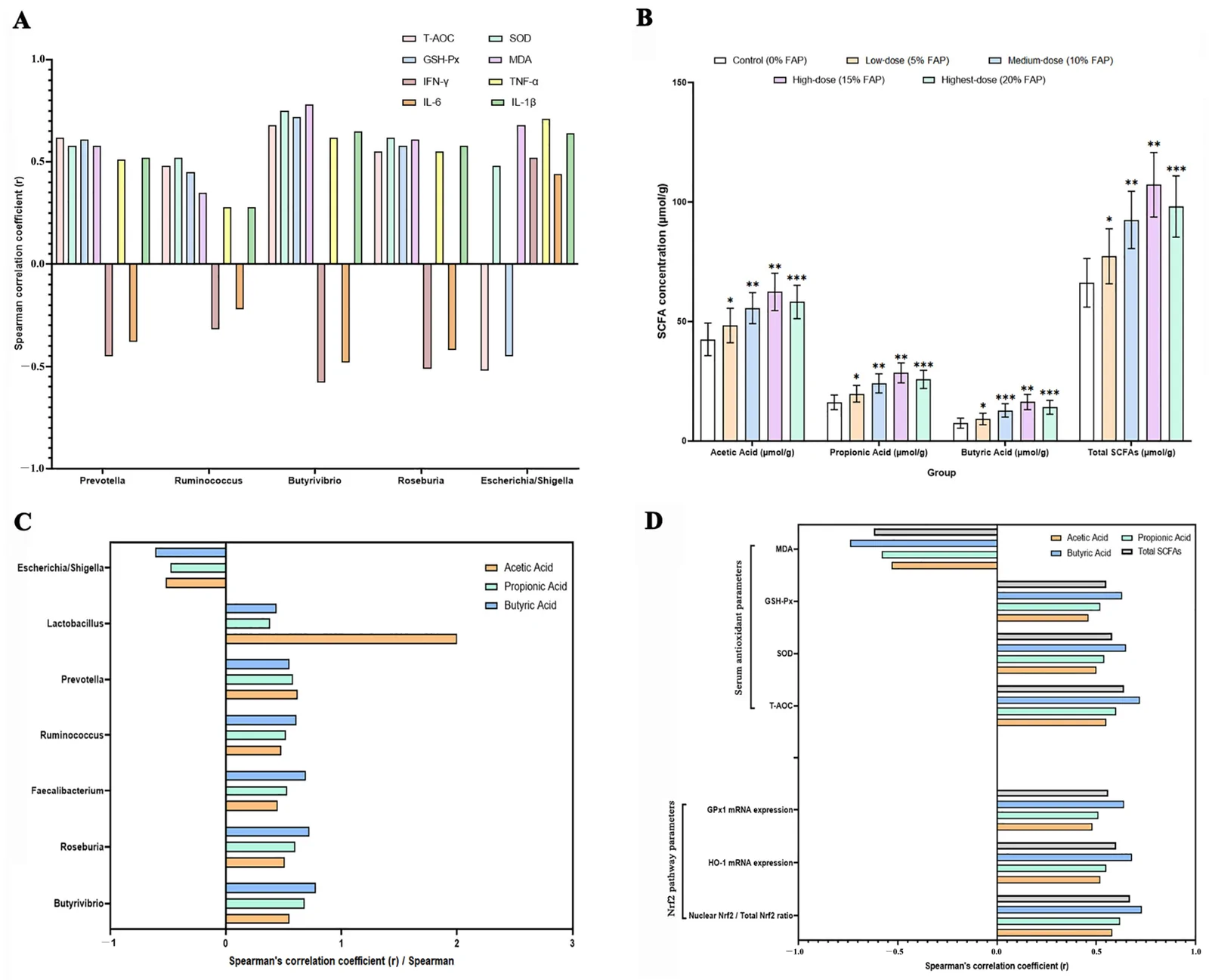
Fermented apple pomace (FAP) modulates cecal short-chain fatty acid (SCFA) production and its associations with gut microbiota and host antioxidant/immune status in rats. (**A**) Spearman correlations between core bacterial genera and serum antioxidant/immune parameters. (**B**) Cecal concentrations of acetic acid, propionic acid, butyric acid, and total SCFAs. (**C**) Spearman correlations between core bacterial genera and SCFAs. (**D**) Spearman correlations between SCFAs and Nrf2 pathway/serum antioxidant parameters. For panels (**A**,**C**,**D**), correlation analyses were based on *n* = 6; no significance markers are shown because no correlation reached the 0.05 significance level (for *n* = 6, |r| ≥ 0.811 corresponds to *p* < 0.05), and these analyses should be considered exploratory and interpreted with caution. For panel (**B**), data are presented as mean ± SD (*n* = 10); * *p* < 0.05, ** *p* < 0.01, *** *p* < 0.001 vs. control (one-way ANOVA followed by Dunnett’s test). T-AOC, total antioxidant capacity; SOD, superoxide dismutase; GSH-Px, glutathione peroxidase; MDA, malondialdehyde; IFN-γ, interferon-γ; TNF-α, tumor necrosis factor-α; IL-6, interleukin-6; IL-1β, interleukin-1β; SCFAs, short-chain fatty acids. The quadratic term was significant for cecal butyric acid and total SCFAs (*p* < 0.05), with estimated peak responses at approximately 15% FAP (Supplementary Table S17).

The genera enriched in the 15% FAP group were closely associated with host antioxidant and immune parameters (Figure 12A; Supplementary Table S13). *Butyrivibrio* showed the strongest numerical positive correlations with serum T-AOC (r = 0.68), SOD (r = 0.75), and GSH-Px (r = 0.72), and negative correlations with MDA (r = −0.78), IFN-γ (r = −0.58), TNF-α (r = −0.62), IL-6 (r = −0.48), and IL-1β (r = −0.65). *Roseburia* and *Prevotella* exhibited similar but somewhat weaker correlation patterns, whereas Escherichia/Shigella showed the opposite trend, with positive correlations with MDA (r = 0.68), TNF-α (r = 0.71), and IL-1β (r = 0.64), and negative correlations with T-AOC (r = −0.52) and SOD (r = −0.48). However, with *n* = 6, none of these correlations reached statistical significance (|r| ≥ 0.811 for *p* < 0.05). These associations should therefore be considered exploratory and interpreted with caution.

Increasing FAP inclusion from 0% to 15% significantly increased cecal acetic acid, propionic acid, butyric acid, and total SCFA concentrations (Figure 12B; Supplementary Table S14). In the 15% FAP group, butyric acid reached its highest value (16.3 ± 3.2 μmol/g), representing a 117.3% increase over the control (7.5 ± 2.1 μmol/g, *p* < 0.001). Acetic acid increased from 42.5 ± 6.8 to 62.4 ± 7.8 μmol/g, propionic acid from 16.2 ± 3.1 to 28.5 ± 4.2 μmol/g, and total SCFAs from 66.2 ± 10.2 to 107.2 ± 13.5 μmol/g (all *p* < 0.0001). At the 20% FAP level, all SCFAs declined relative to the 15% group (butyric acid, 14.1 ± 2.9 μmol/g; total SCFAs, 98.1 ± 12.8 μmol/g), confirming an inverted U-shaped dose–response relationship and identifying 15% FAP as the inclusion level associated with the most favorable responses among the tested levels for FAP-promoted intestinal SCFA production in rats.

Spearman correlation analysis in the 15% FAP group revealed that butyrate-producing genera showed positive numerical correlations with butyric acid concentration (Figure 12C; Supplementary Table S15). *Butyrivibrio* exhibited the highest correlation coefficient with butyric acid (r = 0.78), followed by *Roseburia* (r = 0.72), *Ruminococcus* (r = 0.61), and *Prevotella* (r = 0.55). In contrast, the opportunistic pathogen Escherichia/Shigella showed a negative numerical correlation with butyric acid (r = −0.61). Similar positive associations were observed between these genera and acetic and propionic acids, although the correlation coefficients were generally lower than those for butyric acid. However, with *n* = 6, none of these correlations reached statistical significance (|r| ≥ 0.811 required for *p* < 0.05). These results suggest that FAP may drive butyric acid biosynthesis by enriching SCFA-producing functional microbiota.

Further analysis of the relationships between SCFAs and key nodes of the Nrf2 pathway and serum antioxidant parameters revealed that butyric acid was the most tightly associated effector molecule among the three SCFAs (Figure 12D; Supplementary Table S16). Butyric acid concentration showed strong numerical positive correlations with the nuclear Nrf2/total Nrf2 ratio (r = 0.73), HO-1 mRNA (r = 0.68), GPx1 mRNA (r = 0.64), serum T-AOC (r = 0.72), SOD (r = 0.65), and GSH-Px (r = 0.63), and a negative numerical correlation with MDA (r = −0.74). Acetic acid, propionic acid, and total SCFAs showed similar but generally weaker numerical correlations with these parameters. However, with *n* = 6, none of these correlations reached statistical significance (|r| ≥ 0.811 required for *p* < 0.05).

Collectively, in rats, 15% FAP was associated with enhanced cecal butyrate production and enrichment of butyrate-producing taxa, particularly *Butyrivibrio* and *Roseburia*. Butyrate showed strong correlations with Nrf2 nuclear translocation and downstream antioxidant parameters, suggesting a potential functional link between gut microbial metabolites and host antioxidant defense. However, these data are correlative; they do not establish that butyrate directly activates Nrf2 or that this axis operates in goats. The reduction in butyrate at the 20% FAP level provides a plausible explanation for the attenuated antioxidant and productive responses at this inclusion level, but direct interventional studies are required to establish causality.

## 4. Discussion

This study identified 15% fermented apple pomace (FAP) as the inclusion level associated with the most favorable responses among the tested levels for dairy goat diets, based on dose–response evidence from production performance and complementary rodent mechanistic data. At this level, FAP was associated with improved growth and lactation performance, rumen fermentation, systemic antioxidant capacity, and immune-inflammatory homeostasis in goats, whereas the 20% level attenuated these benefits. The following discussion integrates findings across four levels—dietary nutritional composition, rumen metabolism and endocrine responses, tissue-level signaling pathways, and the gut microbiota–metabolite–host axis—to delineate the functional basis and underlying logic of 15% FAP supplementation.

From a nutritional standpoint, FAP inclusion progressively altered dietary crude protein and fiber contents. Raw apple pomace contained only 4.23% crude protein, whereas fermentation increased the crude protein content of apple pomace, as indicated by the linear increase in dietary CP from 16.50% to 16.86% as FAP inclusion increased from 0% to 20% (Supplementary Table S5). This increase may result from microbial biomass protein synthesis; however, residual non-protein nitrogen from urea and ammonium sulfate added during fermentation may also contribute. Fractionation of true protein versus non-protein nitrogen, together with residual urea/ammonium determination, is required before FAP protein quality can be fully evaluated. Because these fractions were not determined in the present study, the observed increase in crude protein should not be interpreted as direct evidence of improved protein quality. As FAP inclusion increased from 0% to 20%, dietary CP, NDF, and EE increased linearly, while the 15% FAP group achieved a favorable balance: EE peaked at 3.73% in the 15% FAP group without the excessive elevation observed at 20% (3.90%) (Supplementary Table S5), and the Ca/P ratio was 2.44, falling within the recommended range of 2.0–2.5:1 for ruminants (Figure 3A). The NDF/CP ratio remained constant across all groups (2.32–2.33), indicating that the fiber-to-protein macro-structure was not markedly altered by FAP substitution of corn and wheat bran, providing a basis for comparability among treatments. Notably, raw apple pomace was rich in aspartic acid (0.720 g/100 g) and glutamic acid (0.518 g/100 g), but deficient in methionine (0.038 g/100 g) and tryptophan (0.050 g/100 g). With FAP supplementation, total dietary amino acid content decreased linearly from 13.820 g/100 g in the control to 12.842 g/100 g in the 20% FAP group (Figure 3B; Supplementary Table S6). Nevertheless, the 15% FAP group maintained a total amino acid content of 13.086 g/100 g, which was 1.9% higher than that of the 20% group. Although several essential amino acids decreased with increasing FAP inclusion, the dietary essential amino acid concentrations in the 15% FAP group were comparable to or above the recommended concentrations for lactating dairy goats (NRC, 2007) [13], which provides nutrient requirements for small ruminants including goats. However, because amino acid requirements depend on milk yield, body weight, and lactation stage, and because true protein and non-protein nitrogen fractions were not separately characterized, this comparison should be interpreted with caution. Moreover, dietary amino acid concentrations alone do not directly establish metabolizable amino acid adequacy. Previous studies have demonstrated that apple pomace is rich in pectic polysaccharides and polyphenols, which serve as fermentation substrates for rumen microorganisms and promote the proliferation of beneficial bacteria [15]. In the present study, the NFC content of raw apple pomace (44.91%) (Table 1) was higher than that of the replaced wheat bran, providing a readily fermentable energy source and establishing the material basis for improved rumen fermentation efficiency and microbial protein synthesis.

At the rumen level, the 15% FAP group exhibited significantly elevated TVFA concentrations and reduced NH_3_-N. The pooled-sample analysis suggested an apparent shift toward propionate-type fermentation; however, because these VFA proportions were obtained from pooled samples without statistical replication, this observation is descriptive only (Figure 5; Supplementary Table S9). Because individual VFA data were obtained from pooled samples without statistical replication, these apparent shifts in acetate, propionate, butyrate, and the acetate-to-propionate ratio should be interpreted as descriptive trends rather than statistically confirmed effects. Since propionate is the primary gluconeogenic precursor in ruminants, its increased supply, if confirmed by individual-animal measurements, could potentially support hepatic glucose production. However, because individual VFA proportions were obtained from pooled samples without statistical replication, this remains a hypothesis and cannot be used to explain the observed changes in serum glucose or blood urea nitrogen. Previous studies have reported that apple pomace silage inclusion in lactating goats increased rumen acetate, propionate, and TVFA levels [16], and that apple pomace modulates the microbiota and increases the propionate ratio in in vitro fermentation models [17]. The present study suggests that FAP supplementation was associated with lower NH_3_-N concentration (NH_3_-N decreased from 12.6 to 8.6 mg/dL, a 31.7% reduction), which is consistent with reduced nitrogen loss through the urea cycle. However, nitrogen retention per se was not directly measured, and nitrogen balance studies are required to confirm this interpretation.

The endocrinological profile further supports this metabolic regulation. The 15% FAP group simultaneously elevated growth hormone (from 3.52 to 4.19 ng/mL) and triiodothyronine (T3, from 1.24 to 1.53 ng/mL) while reducing insulin to its lowest level (from 12.4 to 8.4 μIU/mL), suggesting changes in circulating insulin and glucose concentrations. However, because insulin sensitivity was not directly measured, this should not be interpreted as direct evidence of improved insulin sensitivity. This endocrine milieu—low insulin, elevated GH, and elevated T3—favors reduced lipolysis and augmented protein synthesis, consistent with the observed decreases in serum triglycerides and total cholesterol (25.8% and 14.8% reductions, respectively). Previous studies in beef cattle have shown that fermented apple pomace-based total mixed rations significantly increased average daily gain and apparent digestibility of crude protein [18]. The present study is the first to systematically describe this endocrine-metabolic pattern in lactating dairy goats. Therefore, we propose that 15% FAP, by modulating dietary energy structure, may influence hepatic gluconeogenic precursor supply and nitrogen metabolism while establishing a hormonally anabolic environment that collectively supports lactation and growth. The pooled VFA analysis showed an apparent increase in propionate proportion at 15% FAP; however, because samples were pooled within treatment, these values should be regarded as descriptive observations and not as treatment effects. Therefore, the apparent shift toward propionate-type fermentation is descriptive only and should not be regarded as a confirmed mechanism. The potential contribution of propionate to the observed metabolic and production responses remains speculative and requires confirmation with individual-animal VFA measurements. The attenuated effects at the 20% level may result from excessive fiber dilution or accumulation of anti-nutritional factors beyond microbial tolerance, thereby compromising fermentation efficiency. An important limitation of the present study is that FAP replaced corn and wheat bran, resulting in diets that were not isonitrogenous or isoenergetic and that differed in CP, NDF, ADF, EE, NFC, and Ca. Consequently, the observed production responses cannot be attributed solely to FAP, and part of the effect may be due to changes in the overall nutrient matrix. This limitation should be considered when interpreting the dose–response results.

At the tissue-signaling level, in rats, 15% FAP was associated with significant suppression of the TLR4/NF-κB pathway, with the p-p65/p65 ratio reduced to 0.12 times that of the control, accompanied by concordant declines in TNF-α and IL-1β at transcriptional, translational, and secretory levels. Apple pomace polyphenols have been demonstrated to exert anti-inflammatory effects by inhibiting NF-κB and COX-2 [19]. The present study provides molecular evidence in a complementary rodent model; validation of this mechanism in dairy goats is needed. Concurrently, FAP decreased Keap1 expression, promoted Nrf2 nuclear translocation (nuclear/total Nrf2 ratio increased by 4.35-fold), and upregulated antioxidant enzymes including HO-1, GPx1, and SOD1, leading to peak serum total antioxidant capacity and a 71.8% reduction in MDA. Polyphenolic compounds in apple pomace have been shown to modulate redox balance through the Nrf2 pathway and regulate inflammatory responses through the NF-κB pathway [20,21]. In the rat model, these two pathways appeared to be coordinately regulated in the 15% FAP group, suggesting that FAP may simultaneously relieve oxidative stress and inflammatory burden, with possible mutual reinforcement: Nrf2-driven antioxidase upregulation scavenges ROS and attenuates redox-sensitive NF-κB activation, while NF-κB suppression curtails inflammation-induced secondary oxidative bursts. However, because these mechanistic data were obtained only in rats, they should be considered supportive and hypothesis-generating rather than as direct evidence of the mechanisms underlying the goat responses.

Notably, IL-6 exhibited an inverse expression pattern relative to other proinflammatory cytokines, being significantly elevated at 15% FAP (30.2 pg/mL vs. 8.5 pg/mL in the control) despite strong upstream NF-κB inhibition. IL-6 has been reported to participate in tissue repair through STAT3 signaling [22,23,24,25,26]; however, because STAT3 activation and tissue-repair markers were not assessed in the present study, this interpretation remains speculative. Further studies measuring phospho-STAT3 and hepatic proliferation markers are required to test this hypothesis.

16S rRNA sequencing revealed that, in rats, 15% FAP significantly increased fecal microbial α-diversity and enriched butyrate-producing genera such as *Butyrivibrio* and *Roseburia*, while suppressing opportunistic pathogens like Escherichia/Shigella (Figure 8, Figure 9 and Figure 10). Concomitantly, cecal butyrate concentration peaked at 15% FAP (16.3 μmol/g, a 117.3% increase over the control) and declined to 14.1 μmol/g at 20% (Figure 12B). Correlation analyses showed strong numerical positive associations between butyrate concentration and Nrf2 nuclear translocation (r = 0.73) as well as serum T-AOC (r = 0.72), and a negative numerical correlation with MDA (r = −0.74) (Figure 12D; Supplementary Table S16). However, with *n* = 6, none of these correlations reached statistical significance (|r| ≥ 0.811 required for *p* < 0.05). These associations should therefore be considered exploratory and interpreted with caution. These statistical associations outline a candidate transmission chain: FAP supplementation → enrichment of butyrate-producing taxa → enhanced butyrate production → Nrf2 activation and antioxidase upregulation → reduced systemic oxidative damage. Because all evidence for this chain is correlative and derived from rats, it should be regarded as a hypothesis-generating framework rather than a proven cascade in goats. Butyrate, as a histone deacetylase (HDAC) inhibitor and GPR109A agonist, has been extensively demonstrated to induce Nrf2 nuclear translocation through GPR109A receptor recognition, thereby encoding antioxidant enzymes for ROS inactivation [27]. Butyrate also reduces reactive oxygen species levels by activating the Nrf2 antioxidant pathway and enhancing glutathione synthesis, alleviating stress damage to the intestinal barrier and immune cells [28]. Furthermore, SCFAs have been shown to modulate Nrf2 redox signaling through specific free fatty acid receptors, with butyrate inducing epigenetic regulation and/or Nrf2 nuclear translocation [29,30,31,32]. Our results align with these mechanisms and provide, in a complementary rodent model, quantitative correlational support for this potential axis. Direct validation in ruminants is still required. Apple pomace pectic polysaccharides have also been shown to significantly promote lactic acid and short-chain fatty acid production in in vitro fermentation [33].

Nevertheless, correlation does not equate to causation. The potential contributions of other microbial metabolites (e.g., propionate, polyphenolic derivatives) cannot be excluded, nor can the dominant signaling route (HDAC inhibition versus GPR109A) be ascertained. The parallel decline in butyrate and Nrf2 activity at the 20% level further reinforces the conclusion that 15% FAP is the inclusion level associated with the most favorable responses among the tested levels, though the underlying cause remains unclear and may involve unidentified antibacterial components in high-dose FAP or reduced substrate accessibility for butyrate producers due to altered fiber structure.

Finally, we acknowledge several limitations. First, the causal inferences regarding the butyrate–Nrf2 axis have not been validated through direct interventions, such as GPR109A antagonists or HDAC inhibitors, nor through Nrf2-knockout models; thus, all mechanistic propositions remain correlative hypotheses. Second, the chemical characterization of bioactive components in FAP—including polyphenols, flavonoids, organic acids, and small peptides—is incomplete, preventing us from distinguishing direct microbiota-modulating agents from host-absorbed compounds. Third, the goat trial covered only 46 days of mid-lactation, without extending to full lactation, reproductive performance, or offspring health, limiting the translational applicability of our findings. Fourth, individual VFA proportions were obtained from pooled rumen samples and therefore lack biological replication; these data are descriptive only and cannot be used to establish causality. Future work should employ intestinal epithelial cell models with exogenous butyrate and specific pathway inhibitors to establish causality, conduct UPLC-MS/MS-based identification of key functional compounds, and perform long-term full-lactation trials with economic analyses to inform practical feeding strategies.

## 5. Conclusions

In summary, 15% FAP was associated with the most favorable production and metabolic responses among the tested inclusion levels in lactating dairy goats, and quadratic dose–response analysis supported a peak response at approximately 15% FAP. However, because FAP replaced corn and wheat bran, the experimental diets were not isonitrogenous or isoenergetic and differed in CP, NDF, ADF, EE, NFC, and Ca. Therefore, part of the observed responses may be attributable to changes in the overall nutrient composition rather than to FAP alone. Future studies using isonitrogenous and isoenergetic diets are needed to isolate the specific effects of FAP. In goats, 15% FAP was accompanied by improved growth and lactation performance, an apparent trend toward propionate-type rumen fermentation based on pooled VFA data, lower ruminal NH_3_-N concentrations, and endocrine changes consistent with an anabolic state. However, nitrogen balance and retention were not directly measured. In rats, 15% FAP was associated with activation of Nrf2 antioxidant signaling, suppression of TLR4/NF-κB inflammation, enrichment of butyrate-producing genera, and elevated cecal butyrate. Butyrate correlated with Nrf2 activation and systemic antioxidant capacity, suggesting a potential microbiota–butyrate–Nrf2–antioxidant axis. However, because hepatic signaling and cecal SCFAs/microbiota were not measured in goats, this axis remains a cross-species hypothesis requiring direct validation in ruminants. All beneficial responses were attenuated at 20% compared with 15%, supporting 15% as the inclusion level associated with the most favorable responses among the tested levels. These findings provide a theoretical basis for precision application of FAP in ruminant nutrition and highlight the gut–host axis as a candidate target for dietary intervention, with butyrate as a critical functional link between microbial remodeling and host defense activation.

## Figures and Tables

**Table 1:** Conventional nutrient and mineral composition of raw apple pomace.

Item	Value
Dry Matter (DM)	89.20 ± 1.47
Crude Protein (CP)	4.23 ± 0.49
Neutral Detergent Fiber (NDF)	44.87 ± 3.56
Acid Detergent Fiber (ADF)	34.52 ± 2.84
Ether Extract (EE)	3.61 ± 0.47
Ash	2.38 ± 0.29
Non-Fiber Carbohydrates (NFC)	44.91 ± 3.12
NDF/CP Ratio	10.61 ± 1.37
Calcium (Ca)	0.12 ± 0.026
Phosphorus (P)	0.08 ± 0.014
Ca/P Ratio	1.50 ± 0.21

Note: DM, dry matter; CP, crude protein; NDF, neutral detergent fiber; ADF, acid detergent fiber; EE, ether extract; NFC, non-fiber carbohydrates; Ca, calcium; P, phosphorus. Values are presented as means ± SD (*n* = 3). All values are expressed on a dry matter (DM) basis, except DM, which is expressed on a fresh weight basis. NFC was calculated as 100 − (CP + NDF + EE + Ash) on a DM basis. NDF/CP and Ca/P are presented as ratios. No statistical comparison was performed because only one group was evaluated; therefore, no *p*-values are reported.

## Data Availability

The original contributions presented in this study are included in the article/Supplementary Materials. Further inquiries can be directed to the corresponding authors.

## Supplementary Materials

The following supporting information can be downloaded at: https://www.mdpi.com/article/10.3390/foods15213770/s1, Table S1: Composition and active ingredient concentrations of the compound vitamin–mineral premix used in the dairy goat diets (per kg of premix); Table S2: Ingredient composition of the experimental total mixed rations for dairy goats containing graded levels of fermented apple pomace (FAP) (% of dietary dry matter); Table S3: Ingredient composition of the AIN-93M purified basal diet and the AIN-93M mineral and vitamin mixes fed to rats (g/kg diet or mix); Table S4: Amino acid composition of raw apple pomace on a dry matter basis (g/100 g DM); Table S5: Effects of graded inclusion levels of fermented apple pomace (FAP) on the nutritional composition of experimental diets for dairy goats (% of dry matter); Table S6: Effects of graded inclusion levels of fermented apple pomace (FAP) on the amino acid composition of experimental diets for dairy goats (g/100 g dry matter); Table S7: Effects of graded inclusion levels of fermented apple pomace (FAP) on growth performance, lactation performance, and milk quality of dairy goats; Table S8: Effects of dietary fermented apple pomace (FAP) supplementation on serum metabolic and hormonal parameters in dairy goats; Table S9: Effects of graded dietary inclusion levels of fermented apple pomace (FAP) on rumen fermentation parameters in lactating dairy goats; Table S10: Effects of graded inclusion levels of fermented apple pomace (FAP) on serum antioxidant indices in rats; Table S11: Effects of graded inclusion levels of fermented apple pomace (FAP) on serum cytokine concentrations in rats; Table S12: Effects of graded inclusion levels of fermented apple pomace (FAP) on alpha diversity of fecal microbiota in rats; Table S13: Spearman correlation coefficients between core gut genera and host antioxidant and immune indices in rats; Table S14: Effects of graded inclusion levels of fermented apple pomace (FAP) on cecal short-chain fatty acid concentrations in rats; Table S15: Spearman correlation coefficients between core gut genera and short-chain fatty acids in the 15% FAP group; Table S16: Spearman correlation coefficients between short-chain fatty acids and the Nrf2 pathway and serum antioxidant indices in rats; Table S17: Linear and quadratic polynomial contrast *p* values for the main outcomes; Figure S1: Representative original Western blot images showing the expression of p-STAT1, STAT1, nuclear Nrf2, total Nrf2, IL-6, IL-1β, GPx1, HO-1, Keap1, and TNF-α in rat liver tissues from fermented apple pomace-treated rats, with β-actin as the loading control. Figure S2: Rarefaction curves of the fecal microbiota in rats. Reference [34] is cited in the Supplementary Materials.

## Abbreviations

The following abbreviations are used in this manuscript:

ADF	acid detergent fiber
ADG	average daily gain
CP	crude protein
DM	dry matter
DMI	dry matter intake
EE	ether extract
FAP	fermented apple pomace
GH	growth hormone
GSH-Px	glutathione peroxidase
HO-1	heme oxygenase-1
IFN-γ	interferon-γ
IL-1β	interleukin-1β
IL-6	interleukin-6
IL-10	interleukin-10
Keap1	Kelch-like ECH-associated protein 1
MDA	malondialdehyde
NDF	neutral detergent fiber
NFC	non-fiber carbohydrates
NF-κB	nuclear factor kappa-light-chain-enhancer of activated B cells
NH_3_-N	ammonia nitrogen
NQO1	NAD(P)H:quinone oxidoreductase 1
Nrf2	nuclear factor erythroid 2-related factor 2
SCC	somatic cell count
SCFAs	short-chain fatty acids
SOD	superoxide dismutase
SSF	solid-state fermentation
T-AOC	total antioxidant capacity
TLR4	Toll-like receptor 4
TNF-α	tumor necrosis factor-α
TVFA	total volatile fatty acids
VFA	volatile fatty acids

## Appendix A

**Table A1:** Ingredient composition and inclusion levels of the total mixed ration (TMR) fed to dairy goats (% of DM).

Items	Content (% of DM)
Concentrate mixture	
Corn	21.8
Soybean meal	3.2
Rapeseed meal	2.4
Peanut meal	2.0
Wheat bran	8.6
Dicalcium phosphate	0.6
Limestone	0.4
Salt	0.4
Sodium bicarbonate	0.2
Premix	0.4
Forage	
Alfalfa hay	25.0
Corn silage	20.0
Chinese wildrye hay	15.0
Total	100.0

Note: Values are expressed as percentages of dietary dry matter (DM). The TMR was formulated with a concentrate-to-forage ratio of 40:60 on a DM basis; the concentrate mixture and forage accounted for 40% and 60% of dietary DM, respectively. Premix, vitamin–mineral premix. The composition of the vitamin–mineral premix is provided in Supplementary Table S1. Abbreviations: DM, dry matter; TMR, total mixed ration.

**Table A2:** Primer sequences used for RT-qPCR analysis of interferon signaling, inflammatory pathways, and the Nrf2 antioxidant pathway.

Gene	Method	Forward Primer (5′→3′)	Reverse Primer (5′→3′)
IFN-γ	Real-time PCR	CCC TCT CTG GCT GTT ACT GC	CTC CTT TTC CGC TTC CTT AG
STAT1	Real-time PCR	TGAGTTCCGACACCTGCAACTGAA	AGGTGGTCTCAAGGTCAATCACCA
IRF1	Real-time PCR	ACGGCTGGGACATCAACAAG	TCCTGCTTTGTATCGGCT
TLR4	Real-time PCR	TCAGTGATTCGGTGGTCAGT	TCCTCATTCTGGCTCAGATA
NF-κB p65	Real-time PCR	AGGCTTCTGGGCCTTATGTG	TGCTTCTCTCGCCAGGAATAC
TNF-α	Real-time PCR	GATCGGTCCCAACAAGGAG	CTTGTCACTCGAGTTTTGAGA
IL-1β	Real-time PCR	ACCTTCCAGGATGAGGACATGA	AACGTCACACACCAGCAGGTTA
IL-6	Real-time PCR	GATACCACCCACAACAGACCA	CAGAATTGCCATTGCACAACTC
IL-10	Real-time PCR	GCTGCCTTCAGTCAAGTGAAG	CTGACAAGGCTTGGCAACC
Keap1	Real-time PCR	CTGGAGGATCATACCAAGCAGG	GGATACCCTCAATGGACACCAC
Nrf2	Real-time PCR	CCCATGTAGGGCTGTGAT	TTGGCGGTTGTTAGGTC
HO-1	Real-time PCR	TAGTTCATCCCAGACACCG	GGCCCAAGAAAAGAGAGCCA
NQO1	Real-time PCR	CAGCGGCTCCATGTACT	GACCTGGAAGCCACAGAAG
GPx1	Real-time PCR	ATAGAAGCCCTGCTGTCCAA	GAAACCGCCTTTCTTTAGGC
SOD1	Real-time PCR	GCGGATGAAGAGAGGCATGTT	ACGGCCAATGATGGAATGCT
CAT	Real-time PCR	ACAACTCCCAGAAGCCTAAGAATG	GCTTTTCCCTTGGCAGCTATG
β-actin	Real-time PCR	TATCGGCAATGAGCGGTTCC	AGCACTGTGTTGGCATAGAGG
