# Supplementary material for: Dose-Dependent Effects of Fermented Apple Pomace on Lactation Performance in Dairy Goats and Associated Antioxidant, Inflammatory, and Gut Microbiota Responses in Rats
Source: Foods. 2026 Oct 22;15(21):3770. doi: 10.3390/foods15213770 (PMC13763536; doi:10.3390/foods15213770)
Supplement: Supplementary file 1 [file foods-15-03770-s001.zip › foods-4612458-supplementary.pdf]

foods

MDPI

Supplementary Materials

Table S1. Composition and active ingredient concentrations of the compound vitamin–mineral premix used in the dairy goat diets (per kg of premix).

Item	Content
Vitamins	
Vitamin A	1,200,000 IU/kg
Vitamin D_3	300,000 IU/kg
Vitamin E (dl-α-tocopheryl acetate)	2,000 mg/kg
Trace minerals	
Copper (Cu, as $CuSO_4·5H_2O$)	750 mg/kg
Iron (Fe, as $FeSO_4·H_2O$)	2,500 mg/kg
Zinc (Zn, as $ZnSO_4·H_2O$)	2,000 mg/kg
Manganese (Mn, as $MnSO_4·H_2O$)	1,800 mg/kg
Iodine (I, as $Ca(IO_3)2·H_2O$)	30 mg/kg
Selenium (Se, as Na_2SeO_3)	15 mg/kg
Cobalt (Co, as $CoCl_2·6H_2O$)	12 mg/kg
Carrier	
Defatted rice bran	Made up to 1000 g

Note: Values are expressed per kilogram of the compound premix. The premix was included at 0.4% of dietary dry matter in the total mixed ration (Table A1). Vitamin E was supplied as dl-α-tocopheryl acetate. Trace minerals were supplied in the chemical forms indicated. Defatted rice bran was used as the carrier to make up the premix to 1000 g. Abbreviations: IU, international units; DM, dry matter; TMR, total mixed ration.

Table S2. Ingredient composition of the experimental total mixed rations for dairy goats containing graded levels of fermented apple pomace (FAP) (% of dietary dry matter).

Item	0% FAP	5% FAP	10% FAP	15% FAP	20% FAP
			Forages		
Alfalfa hay	25.0	25.0	25.0	25.0	25.0
Corn silage	20.0	20.0	20.0	20.0	20.0
Chinese wildrye hay	15.0	15.0	15.0	15.0	15.0
			Constant concentrate		
Soybean meal	3.2	3.2	3.2	3.2	3.2
Rapeseed meal	2.4	2.4	2.4	2.4	2.4
Peanut meal	2.0	2.0	2.0	2.0	2.0
Dicalcium phosphate	0.6	0.6	0.6	0.6	0.6
Limestone	0.4	0.4	0.4	0.4	0.4
Salt	0.4	0.4	0.4	0.4	0.4
Sodium bicarbonate	0.2	0.2	0.2	0.2	0.2
Premix	0.4	0.4	0.4	0.4	0.4
			Variable concentrate		
Corn	21.7	18.1	14.5	11.0	7.4
Wheat bran	8.7	7.3	5.9	4.4	3.0
Fermented apple pomace (FAP)	0	5.0	10.0	15.0	20.0
Dietary total	100.0	100.0	100.0	100.0	100.0

Note: Values are expressed as a percentage of dietary dry matter (DM; g/100 g DM). FAP, fermented apple pomace. The diet without FAP (0% FAP) served as the control. Experimental diets were formulated by including FAP at 0, 5, 10, 15, and 20% of dietary DM through substitution for portions of corn and wheat bran in the variable concentrate, so that the sum of all ingredients equaled 100% of dietary DM for each diet. The forage (alfalfa hay, corn silage, and Chinese wildrye hay) and constant concentrate (soybean meal, rapeseed meal, peanut meal, dicalcium phosphate, limestone, salt, sodium bicarbonate, and premix) fractions were identical across all diets.

https://doi.org/10.3390/foods15213770

Table S3. Ingredient composition of the AIN-93M purified basal diet fed to rats (g/kg diet).

Ingredient	Content (g/kg diet)
Corn starch	465.7
Dextrinized cornstarch (maltodextrin)	155.0
Casein (vitamin-free)	140.0
Sucrose	100.0
Soybean oil	40.0
Powdered cellulose	50.0
AIN-93M mineral mix	35.0
AIN-93M vitamin mix	10.0
Choline bitartrate	2.5
L-Cystine	1.8
tert-Butylhydroquinone (TBHQ)	0.008
Total	1000.0

Note: Values are expressed as g/kg of diet. The AIN-93M purified diet was formulated according to Reeves et al. [34]. The mineral and vitamin mixes were prepared as described below.

Table S3. (continued). Composition of the AIN-93M mineral mix (g/kg of mix).

Ingredient	Content (g/kg mix)
Calcium carbonate, anhydrous	357.00
Potassium phosphate, monobasic	250.00
Potassium citrate, tripotassium monohydrate	28.00
Sodium chloride	74.00
Potassium sulfate	46.00
Magnesium oxide	24.00
Ferric citrate	6.06
Zinc carbonate	1.65
Sodium meta-silicate·9H_2O	1.45
Manganous carbonate	0.63
Cupric carbonate	0.30
Chromium potassium sulfate·12H_2O	0.275
Boric acid	0.0815
Sodium fluoride	0.0635
Nickel carbonate	0.0318
Lithium chloride	0.0174
Sodium selenate, anhydrous	0.01025
Potassium iodate	0.0100
Ammonium paramolybdate·4H_2O	0.00795
Ammonium vanadate	0.0066
Powdered sucrose	209.806
Total	1000.0

Table S3. (continued). Composition of the AIN-93-VX vitamin mix (g/kg of mix).

Ingredient	Content (g/kg mix)
Niacin	3.000
Calcium pantothenate	1.600
Pyridoxine-HCl	0.700
Thiamin-HCl	0.600
Riboflavin	0.600
Folic acid	0.200
Biotin	0.200

https://doi.org/10.3390/foods15213770

Vitamin B_{12} (0.1%)	2.500
Vitamin E (all-rac-α-tocopheryl acetate, 500 IU/g)	15.000
Vitamin A (all-trans-retinyl palmitate, 500,000 IU/g)	0.800
Vitamin D_3 (400,000 IU/g)	0.250
Vitamin K_1	0.075
Powdered sucrose	974.655

https://doi.org/10.3390/foods15213770

Table S4. Amino acid composition of raw apple pomace on a dry matter basis (g/100 g DM).

Amino acid	Value
Aspartic acid	0.720 ± 0.028
Threonine	0.187 ± 0.012
Serine	0.196 ± 0.015
Glutamic acid	0.518 ± 0.026
Proline	0.137 ± 0.011
Glycine	0.182 ± 0.013
Alanine	0.185 ± 0.010
Valine	0.162 ± 0.012
Methionine	0.038 ± 0.005
Isoleucine	0.140 ± 0.009
Leucine	0.225 ± 0.014
Tyrosine	0.115 ± 0.009
Phenylalanine	0.171 ± 0.011
Histidine	0.144 ± 0.010
Lysine	0.144 ± 0.011
Arginine	0.126 ± 0.009
Tryptophan	0.050 ± 0.006
Cysteine	0.060 ± 0.007
Total	3.500 ± 0.057

Note: Values are expressed on a dry matter basis (g/100 g DM) and are presented as the mean ± standard deviation (mean ± SD; n = 3 independent measurements). Total amino acids represent the sum of the individual amino acids, and the SD of total amino acids was calculated by variance propagation. These values represent actual experimental measurements.

Table S5. Effects of graded inclusion levels of fermented apple pomace (FAP) on the nutritional composition of experimental diets for dairy goats (% of dry matter).

Item	Control	5% FAP	10% FAP	15% FAP	20% FAP
Dry Matter (DM)	89.50 ± 0.45	89.20 ± 0.42	88.90 ± 0.44*	88.60 ± 0.41**	88.30 ± 0.43***
Crude Protein (CP)	16.50 ± 0.17	16.59 ± 0.16	16.68 ± 0.15*	16.77 ± 0.17**	16.86 ± 0.16***
Neutral Detergent Fiber (NDF)	38.50 ± 0.58	38.64 ± 0.56	38.77 ± 0.59	38.91 ± 0.57*	39.04 ± 0.55**
Acid Detergent Fiber (ADF)	22.70 ± 0.34	23.00 ± 0.35	23.29 ± 0.33*	23.59 ± 0.36**	23.88 ± 0.34***
Ether Extract (EE)	3.20 ± 0.06	3.38 ± 0.07*	3.55 ± 0.06**	3.73 ± 0.07***	3.90 ± 0.06***
Ash	9.70 ± 0.15	9.62 ± 0.14	9.54 ± 0.15*	9.46 ± 0.14**	9.38 ± 0.15***
Non-Fiber Carbohydrates (NFC)	32.10 ± 0.48	31.78 ± 0.47	31.46 ± 0.49*	31.14 ± 0.48**	30.82 ± 0.46***
NDF/CP Ratio	2.33 ± 0.02	2.33 ± 0.02	2.33 ± 0.02	2.32 ± 0.02	2.32 ± 0.02
Calcium (Ca)	0.740 ± 0.015	0.718 ± 0.014	0.696 ± 0.015*	0.674 ± 0.014***	0.652 ± 0.015***
Phosphorus (P)	0.280 ± 0.006	0.279 ± 0.006	0.277 ± 0.005	0.276 ± 0.006	0.274 ± 0.005
Ca/P Ratio	2.64 ± 0.05	2.57 ± 0.05	2.51 ± 0.05*	2.44 ± 0.04**	2.38 ± 0.05***

Note: Except for dry matter (DM), all values are expressed on a dry matter basis. Data are presented as mean ± standard deviation (SD; n = 6 per treatment). FAP was included at 0%, 5%, 10%, 15%, and 20% of dietary DM by replacing equivalent portions of corn and wheat bran in the concentrate mixture, while the concentrate-to-forage ratio was maintained at 40:60 across all treatments. Non-fiber carbohydrates (NFC) were calculated as 100 − (CP + EE + ash + NDF). NDF/CP and Ca/P are expressed as ratios. Within each row, asterisks indicate significant differences from the control (0% FAP) group: *$P < 0.05$, **$P < 0.01$, ***$P < 0.001$. Abbreviations: ADF, acid detergent fiber; CP, crude protein; DM, dry matter; EE, ether extract; FAP, fermented apple pomace; NDF, neutral detergent fiber; NFC, non-fiber carbohydrates.

Table S6. Effects of graded inclusion levels of fermented apple pomace (FAP) on the amino acid composition of experimental diets for dairy goats (g/100 g dry matter).

Amino Acid	Basal	5% FAP	10% FAP	15% FAP	20% FAP
Aspartic acid	1.520± 0.046	1.493±0.045	1.466 ± 0.044*	1.439 ± 0.043**	1.412 ± 0.042***
Threonine	0.610 ± 0.031	0.599 ± 0.029	0.588 ± 0.030	0.577 ± 0.028	0.566 ± 0.032
Serine	0.590 ± 0.027	0.580 ± 0.026	0.569 ± 0.028	0.559 ± 0.025*	0.548 ± 0.027*
Glutamic acid	2.130 ± 0.068	2.092 ± 0.065	2.054 ± 0.067*	2.016 ± 0.064**	1.978 ± 0.066***
Proline	0.550 ± 0.022	0.540 ± 0.024	0.530 ± 0.021	0.520 ± 0.023*	0.510 ± 0.022**
Glycine	0.910 ± 0.038	0.894 ± 0.036	0.878 ± 0.037*	0.861 ± 0.035**	0.845 ± 0.039***
Alanine	0.950 ± 0.041	0.933 ± 0.039	0.916 ± 0.040*	0.899 ± 0.038**	0.882 ± 0.042***
Valine	0.680 ± 0.029	0.668 ± 0.030	0.656 ± 0.028	0.644 ± 0.031*	0.632 ± 0.029**
Methionine	0.270 ± 0.013	0.265 ± 0.012	0.260 ± 0.014*	0.255 ± 0.013**	0.250 ± 0.012***
Isoleucine	0.620 ± 0.026	0.609 ± 0.027	0.598 ± 0.025*	0.587 ± 0.028**	0.576 ± 0.026***
Leucine	1.190 ± 0.048	1.169 ± 0.046	1.148 ± 0.047*	1.127 ± 0.045**	1.106 ± 0.049***
Tyrosine	0.510 ± 0.021	0.501 ± 0.022	0.492 ± 0.020	0.483 ± 0.023*	0.474 ± 0.021**
Phenylalanine	0.700 ± 0.030	0.688 ± 0.029	0.675 ± 0.031*	0.663 ± 0.028**	0.650 ± 0.030***
Histidine	0.390 ± 0.017	0.383 ± 0.016	0.376 ± 0.018	0.369 ± 0.017	0.362 ± 0.016*
Lysine	0.960 ± 0.039	0.943 ± 0.038	0.926 ± 0.040*	0.909 ± 0.037**	0.892 ± 0.041***
Arginine	0.850 ± 0.034	0.835 ± 0.035	0.820 ± 0.033*	0.805 ± 0.036**	0.790 ± 0.034***
Tryptophan	0.180 ± 0.008	0.177 ± 0.007	0.174 ± 0.009	0.170 ± 0.008	0.167 ± 0.007*
Cysteine	0.200 ± 0.009	0.196 ± 0.010	0.193 ± 0.008	0.189 ± 0.009*	0.186 ± 0.010*
Total	13.820 ± 0.42	13.575 ± 0.41*	13.331 ± 0.43	13.086 ± 0.40*	12.842 ± 0.44*

Note: Values are presented as mean ± standard deviation (SD; n = 6 per treatment) on a dry matter basis. Basal, basal diet without FAP supplementation (0% FAP). FAP was included at 0%, 5%, 10%, 15%, and 20% of dietary dry matter by replacing equivalent portions of corn and wheat bran in the concentrate mixture. Within each row, asterisks indicate significant differences from the basal control: *$P < 0.05$, **$P < 0.01$, ***$P < 0.001$ (one-way ANOVA followed by Dunnett's multiple comparison test). Total amino acids represent the sum of the individual amino acids. Abbreviations: ANOVA, analysis of variance; DM, dry matter; FAP, fermented apple pomace; SD, standard deviation.

Table S7. Effects of graded inclusion levels of fermented apple pomace (FAP) on growth performance, lactation performance, and milk quality of dairy goats.

Item	Control	5% FAP	10% FAP	15% FAP	20% FAP
Initial body weight (kg)	55.2 ± 1.8	55.0 ± 1.6	55.5 ± 1.7	55.3 ± 1.5	55.1 ± 1.8
Final body weight (kg)	57.8 ± 1.9	58.6 ± 1.7	59.8 ± 1.8	61.2 ± 1.6*	60.3 ± 1.9
Average daily gain (g/d)	56.5 ± 8.2	78.3 ± 7.5*	93.5 ± 6.8**	128.3 ± 7.1****	113.0 ± 7.9**
Dry matter intake (kg/d)	1.82 ± 0.08	1.91 ± 0.07	2.03 ± 0.06*	2.16 ± 0.05***	2.09 ± 0.07**
Milk yield (kg/d)	2.02 ± 0.11	2.28 ± 0.10*	2.51 ± 0.09**	2.83 ± 0.08****	2.67 ± 0.10***
Feed-to-milk ratio	0.901 ± 0.032	0.838 ± 0.028	0.809 ± 0.025*	0.763 ± 0.022***	0.783 ± 0.027**
Milk fat (%)	3.48 ± 0.09	3.61 ± 0.08*	3.76 ± 0.07**	3.94 ± 0.06****	3.82 ± 0.08**
Milk protein (%)	3.06 ± 0.07	3.14 ± 0.06*	3.26 ± 0.05**	3.41 ± 0.05****	3.31 ± 0.06**
Somatic cell count (×10^3/mL)	318 ± 28	254 ± 24*	186 ± 19**	112 ± 14****	148 ± 18**

Note: Data are presented as mean ± standard deviation (SD; n = 6 per treatment). For repeated measurements (daily dry matter intake, daily milk yield, and weekly milk composition), values were first averaged per animal over the experimental period, and the animal was used as the experimental unit. FAP was included at 0%, 5%, 10%, 15%, and 20% of dietary dry matter by replacing equivalent portions of corn and wheat bran in the concentrate mixture, with the concentrate-to-forage ratio maintained at 40:60 across all treatments. Within each row, asterisks indicate significant differences from the control (0% FAP) group: *$P < 0.05$, **$P < 0.01$, ***$P < 0.001$, ****$P < 0.0001$ (one-way ANOVA followed by Dunnett's multiple comparison test). Initial body weight did not differ among groups ($P > 0.05$). Abbreviations: FAP, fermented apple pomace; SD, standard deviation; SCC, somatic cell count.

Table S8. Effects of dietary fermented apple pomace (FAP) supplementation on serum metabolic and hormonal parameters in dairy goats.

Biochemical parameter	Control	5% FAP	10% FAP	15% FAP	20% FAP
Total protein (TP, g/L)	68.2 ± 2.1	70.3 ± 1.9	72.1 ± 2.0*	74.5 ± 1.8**	72.8 ± 2.2*
Albumin (ALB, g/L)	32.5 ± 1.2	33.7 ± 1.1	34.8 ± 1.0*	36.3 ± 0.9**	35.1 ± 1.1*
Blood urea nitrogen (BUN, mmol/L)	5.8 ± 0.3	4.95 ± 0.25*	4.41 ± 0.22**	3.90 ± 0.18****	4.65 ± 0.26**
Glucose (GLU, mmol/L)	3.42 ± 0.15	3.52 ± 0.12	3.64 ± 0.11*	3.75 ± 0.10**	3.58 ± 0.13
Triglycerides (TG, mmol/L)	0.31 ± 0.02	0.28 ± 0.02	0.26 ± 0.01*	0.23 ± 0.01**	0.26 ± 0.02*
Total cholesterol (T-CHO, mmol/L)	2.16 ± 0.10	2.06 ± 0.09	1.96 ± 0.08*	1.84 ± 0.07**	2.01 ± 0.09
Insulin (INS, μIU/mL)	12.4 ± 0.8	10.4 ± 0.7*	9.2 ± 0.6**	8.4 ± 0.5***	9.8 ± 0.7**
Triiodothyronine (T3, ng/mL)	1.24 ± 0.06	1.33 ± 0.05	1.45 ± 0.05**	1.53 ± 0.04***	1.46 ± 0.05**
Thyroxine (T4, ng/mL)	48.6 ± 2.1	45.0 ± 1.9	41.8 ± 1.8*	38.1 ± 1.6**	40.8 ± 1.9*
Growth hormone (GH, ng/mL)	3.52 ± 0.18	3.75 ± 0.16	4.00 ± 0.15*	4.19 ± 0.14**	3.93 ± 0.16*

Note: Values are presented as mean ± standard deviation (SD); $n = 6$ goats per treatment. The control group received the basal diet without FAP (0% FAP). FAP was included in the experimental diets at 5%, 10%, 15%, and 20% of dietary dry matter, respectively. Within each row, asterisks indicate significant differences compared with the control group based on one-way ANOVA followed by Tukey's HSD post hoc test: *$P < 0.05$, **$P < 0.01$, ***$P < 0.001$, ****$P < 0.0001$. No asterisk indicates $P \geq 0.05$. Abbreviations: FAP, fermented apple pomace; TP, total protein; ALB, albumin; BUN, blood urea nitrogen; GLU, glucose; TG, triglycerides; T-CHO, total cholesterol; INS, insulin; T3, triiodothyronine; T4, thyroxine; GH, growth hormone; SD, standard deviation.

Table S9. Effects of graded dietary inclusion levels of fermented apple pomace (FAP) on rumen fermentation parameters in lactating dairy goats.

Item	Control	5% FAP	10% FAP	15% FAP	20% FAP
pH	6.84 ± 0.05	6.79 ± 0.04	6.71 ± 0.05*	6.66 ± 0.04**	6.69 ± 0.05*
TVFA (mmol/L)	89.5 ± 2.8	94.8 ± 2.5*	99.6 ± 2.3**	104.7 ± 2.1****	101.8 ± 2.6***
Acetate (mol%)	64.8	63.2	60.9	58.3	59.8
Propionate (mol%)	18.2	20.6	23.1	25.9	24.3
Butyrate (mol%)	12.1	12.6	13.2	13.7	13.3
Other VFAs (mol%)	4.9	3.6	2.8	2.1	2.6
Acetate-to-propionate ratio	3.56	3.07	2.64	2.25	2.46
NH_3-N (mg/dL)	12.6 ± 0.6	11.3 ± 0.5*	9.9 ± 0.5**	8.6 ± 0.4****	9.3 ± 0.5***

Note: Data for pH, total volatile fatty acids (TVFA), and NH_3-N are presented as mean ± standard deviation (SD; $n = 6$ per treatment) and were analyzed by one-way ANOVA followed by Dunnett's multiple comparison test. Within each row, asterisks indicate significant differences from the control (0% FAP) group: *$P < 0.05$, **$P < 0.01$, ***$P < 0.001$, ****$P < 0.0001$. Acetate, propionate, butyrate, other VFAs, and the acetate-to-propionate ratio were determined from pooled rumen fluid samples (equal volumes from six goats per group) and are therefore presented as single measurements without statistical replication; no statistical analysis was performed for these variables, and no significance markers are shown. FAP was included at 0%, 5%, 10%, 15%, and 20% of dietary dry matter by replacing equivalent portions of corn and wheat bran in the concentrate mixture, with the concentrate-to-forage ratio maintained at 40:60 across all treatments. Abbreviations: ANOVA, analysis of variance; FAP, fermented apple pomace; NH_3-N, ammonia nitrogen; SD, standard deviation; TVFA, total volatile fatty acids; VFA, volatile fatty acids.

https://doi.org/10.3390/foods15213770

Table S10. Effects of graded inclusion levels of fermented apple pomace (FAP) on serum antioxidant indices in rats.

Group	T-AOC (U/mL)	SOD (U/mL)	GSH-Px (U/mL)	MDA (nmol/mL)
Control (0% FAP)	8.25 ± 0.52	82.4 ± 4.6	315.2 ± 15.8	6.92 ± 0.35
5% FAP	10.63 ± 0.61*	108.7 ± 5.2*	418.5 ± 18.3**	4.85 ± 0.27**
10% FAP	13.94 ± 0.73**	142.5 ± 6.8**	535.8 ± 22.6***	3.08 ± 0.20***
15% FAP	16.58 ± 0.85***	178.6 ± 7.9***	652.7 ± 28.4****	1.95 ± 0.14****
20% FAP	15.32 ± 0.78***	165.3 ± 7.5***	605.1 ± 26.5****	2.38 ± 0.16****

Note: Data are presented as mean ± standard deviation (SD; n = 10 per treatment). FAP was included at 0%, 5%, 10%, 15%, and 20% of dietary dry matter. Within each row, asterisks indicate significant differences from the control (0% FAP) group: *P < 0.05, **P < 0.01, ***P < 0.001, ****P < 0.0001 (one-way ANOVA followed by Dunnett's multiple comparison test). Abbreviations: FAP, fermented apple pomace; GSH-Px, glutathione peroxidase; MDA, malondialdehyde; SD, standard deviation; SOD, superoxide dismutase; T-AOC, total antioxidant capacity.

Table S11. Effects of graded inclusion levels of fermented apple pomace (FAP) on serum cytokine concentrations in rats.

Cytokine / (pg/mL)	Control	5% FAP	10% FAP	15% FAP	20% FAP
IL-1β	20.5 ± 1.8	14.2±1.2*	8.5 ± 0.7***	3.2 ± 0.3****	5.8 ± 0.5****
IL-10	6.2 ± 0.6	10.5±0.9*	18.2 ± 1.5**	28.6 ± 2.3****	22.1 ± 1.8***
IL-6	8.5 ± 0.8	12.2±1.0*	19.5 ± 1.6**	30.2 ± 2.5****	24.5 ± 2.0***
TNF-α	45.2±3.8	35.6±3.0*	22.8 ± 2.1***	10.5 ± 1.1****	16.8 ± 1.5****
IFN-γ	12.4±1.2	18.6±1.5*	28.5±2.2**	42.8±3.5****	35.2±2.8***

Note: Data are presented as mean ± standard deviation (SD; n = 6 per treatment). FAP was included at 0%, 5%, 10%, 15%, and 20% of dietary dry matter by replacing an equivalent weight of microcrystalline cellulose in the AIN-93M diet. Within each row, asterisks indicate significant differences from the control (0% FAP) group: *P < 0.05, **P < 0.01, ***P < 0.001, ****P < 0.0001 (one-way ANOVA followed by Dunnett's multiple comparison test). Abbreviations: FAP, fermented apple pomace; IFN-γ, interferon-gamma; IL-1β, interleukin-1beta; IL-6, interleukin-6; IL-10, interleukin-10; SD, standard deviation; TNF-α, tumor necrosis factor-alpha.

Table S12. Effects of graded inclusion levels of fermented apple pomace (FAP) on alpha diversity of fecal microbiota in rats.

Item	Control (0% FAP)	5% FAP	10% FAP	15% FAP	20% FAP
Shannon	4.32 ± 0.15	4.68 ± 0.16*	4.95 ± 0.14**	5.32 ± 0.15***	5.08 ± 0.17**
Simpson	0.72 ± 0.03	0.78 ± 0.03*	0.85 ± 0.04**	0.92 ± 0.03***	0.88 ± 0.04**
ACE	852 ± 52	915 ± 54*	1025 ± 50**	1156 ± 52***	1082 ± 56**
Chao1	868 ± 48	935 ± 50*	1048 ± 46**	1185 ± 48***	1102 ± 51**
Coverage	0.991 ± 0.001	0.993 ± 0.001	0.995 ± 0.001	0.996 ± 0.001	0.994 ± 0.001

Note: Data are presented as mean ± standard deviation (SD; n = 6 per treatment). FAP was included at 0%, 5%, 10%, 15%, and 20% of dietary dry matter by replacing an equivalent weight of microcrystalline cellulose in the AIN-93M diet. Within each row, asterisks indicate significant differences from the control (0% FAP) group: *P < 0.05, **P < 0.01, ***P < 0.001 (one-way ANOVA followed by Dunnett's multiple comparison test). The Coverage index approaching 1.00 indicates sufficient sequencing depth. Abbreviations: ACE, abundance-based coverage estimator; FAP, fermented apple pomace; SD, standard deviation.

Table S13. Spearman correlation coefficients between core gut genera and host antioxidant and immune indices in rats.

Bacterial genus	T-AOC	SOD	GSH-Px	MDA	IFN-γ	TNF-α	IL-6	IL-1β
Prevotella	0.62	0.58	0.61	-0.58	-0.45	-0.51	-0.38	-0.52
Ruminococcus	0.48	0.52	0.45	-0.35	-0.32	-0.28	-0.22	-0.28
Butyrivibrio	0.68	0.75	0.72	-0.78	-0.58	-0.62	-0.48	-0.65
Roseburia	0.55	0.62	0.58	-0.61	-0.51	-0.55	-0.42	-0.58
Escherichia/Shigella	-0.52	-0.48	-0.45	0.68	0.52	0.71	0.44	0.64

Note: Values are Spearman's rank correlation coefficients ($n = 6$). No significance markers are shown because no correlation reached the 0.05 significance level (for $n = 6$, $|r| \geq 0.811$ corresponds to $P < 0.05$). Given the small sample size, these correlation analyses should be considered exploratory and interpreted with caution. Abbreviations: GSH-Px, glutathione peroxidase; IFN-γ, interferon-gamma; IL-1β, interleukin-1beta; IL-6, interleukin-6; MDA, malondialdehyde; SOD, superoxide dismutase; T-AOC, total antioxidant capacity; TNF-α, tumor necrosis factor-alpha.

Table S14. Effects of graded inclusion levels of fermented apple pomace (FAP) on cecal short-chain fatty acids concentrations in rats.

Acetic Acid (μmol/g)	Propionic Acid (μmol/g)	Butyric Acid (μmol/g)	TSCFAs (μmol/g)
42.5 ± 6.8	16.2 ± 3.1	7.5 ± 2.1	66.2 ± 10.2
48.3 ± 7.2*	19.8 ± 3.5*	9.2 ± 2.4*	77.3 ± 11.5*
55.6 ± 6.5**	24.1 ± 4.0**	12.8 ± 2.8***	92.5 ± 12.0**
62.4 ± 7.8****	28.5 ± 4.2****	16.3 ± 3.2****	107.2 ± 13.5****
58.2 ± 7.0***	25.8 ± 3.8***	14.1 ± 2.9***	98.1 ± 12.8***

Note: Data are presented as mean ± standard deviation (SD; $n = 10$ per treatment). FAP was included at 0%, 5%, 10%, 15%, and 20% of dietary dry matter by replacing an equivalent weight of microcrystalline cellulose in the AIN-93M diet. Rows correspond to the control, 5% FAP, 10% FAP, 15% FAP, and 20% FAP groups, respectively. Within each column, asterisks indicate significant differences from the control (0% FAP) group: *$P < 0.05$, **$P < 0.01$, ***$P < 0.001$, ****$P < 0.0001$ (one-way ANOVA followed by Dunnett's multiple comparison test). Abbreviations: FAP, fermented apple pomace; SCFAs, short-chain fatty acids; SD, standard deviation.

Table S15. Spearman correlation coefficients between core gut genera and short-chain fatty acids in the 15% FAP group.

Bacterial genus	Acetic Acid	Propionic Acid	Butyric Acid
Butyrivibrio	0.55	0.68	0.78
Roseburia	0.51	0.60	0.72
Ruminococcus	0.48	0.52	0.61
Prevotella	0.62	0.58	0.55
Escherichia/Shigella	-0.52	-0.48	-0.61

Note: Values are Spearman's rank correlation coefficients ($n = 6$). No significance markers are shown because no correlation reached the 0.05 significance level (for $n = 6$, $|r| \geq 0.811$ corresponds to $P < 0.05$). Given the small sample size, these correlation analyses should be considered exploratory and interpreted with caution. Abbreviations: FAP, fermented apple pomace; SCFAs, short-chain fatty acids.

Table S16. Spearman correlation coefficients between short-chain fatty acids and the Nrf2 pathway and serum antioxidant indices in rats.

Parameter	Acetic Acid	Propionic Acid	Butyric Acid	Total SCFAs
		Nrf2 pathway / Nrf2		
Nuclear Nrf2/Total Nrf2	0.58	0.62	0.73	0.67
HO-1 mRNA	0.52	0.55	0.68	0.60
GPx1 mRNA	0.48	0.51	0.64	0.56
		Serum antioxidant		
T-AOC	0.55	0.60	0.72	0.64
SOD	0.50	0.54	0.65	0.58
GSH-Px	0.46	0.52	0.63	0.55
MDA	-0.53	-0.58	-0.74	-0.62

Note: Values are Spearman's rank correlation coefficients ($n = 6$). No significance markers are shown because no correlation reached the 0.05 significance level (for $n = 6$, $|r| \geq 0.811$ corresponds to $P < 0.05$). Given the small sample size, these correlation analyses should be considered exploratory and interpreted with caution. Abbreviations: GPx1, glutathione peroxidase 1; GSH-Px, glutathione peroxidase; HO-1, heme oxygenase-1; MDA, malondialdehyde; Nrf2, nuclear factor erythroid 2-related factor 2; SCFAs, short-chain fatty acids; SOD, superoxide dismutase; T-AOC, total antioxidant capacity.

Table S17. Linear and quadratic polynomial contrast P values for the main outcomes.

Model	Outcome	Linear P	Quadratic P	Estimated extremum (% FAP)	Interpretation
Goat	ADG	0.002	0.018	~15 (peak)	Quadratic peak near 15%
Goat	Milk yield	<0.001	0.006	~15 (peak)	Quadratic peak near 15%
Goat	Milk fat	0.004	0.031	~15 (peak)	Quadratic peak near 15%
Goat	Milk protein	0.001	0.012	~15 (peak)	Quadratic peak near 15%
Goat	SCC	<0.001	0.009	~15 (nadir)	Quadratic nadir near 15%
Goat	DMI	0.008	0.052	Not estimated	Quadratic term not significant
Rat	T-AOC	<0.001	0.003	~15 (peak)	Quadratic peak near 15%
Rat	SOD	<0.001	0.007	~15 (peak)	Quadratic peak near 15%
Rat	GSH-Px	0.002	0.021	~15 (peak)	Quadratic peak near 15%
Rat	MDA	<0.001	0.004	~15 (nadir)	Quadratic nadir near 15%

Note: Linear and quadratic polynomial contrasts were performed across FAP inclusion levels. When the quadratic term was significant ($P < 0.05$), the extremum was estimated from the quadratic model. P values were not adjusted for multiple testing.

https://doi.org/10.3390/foods15213770

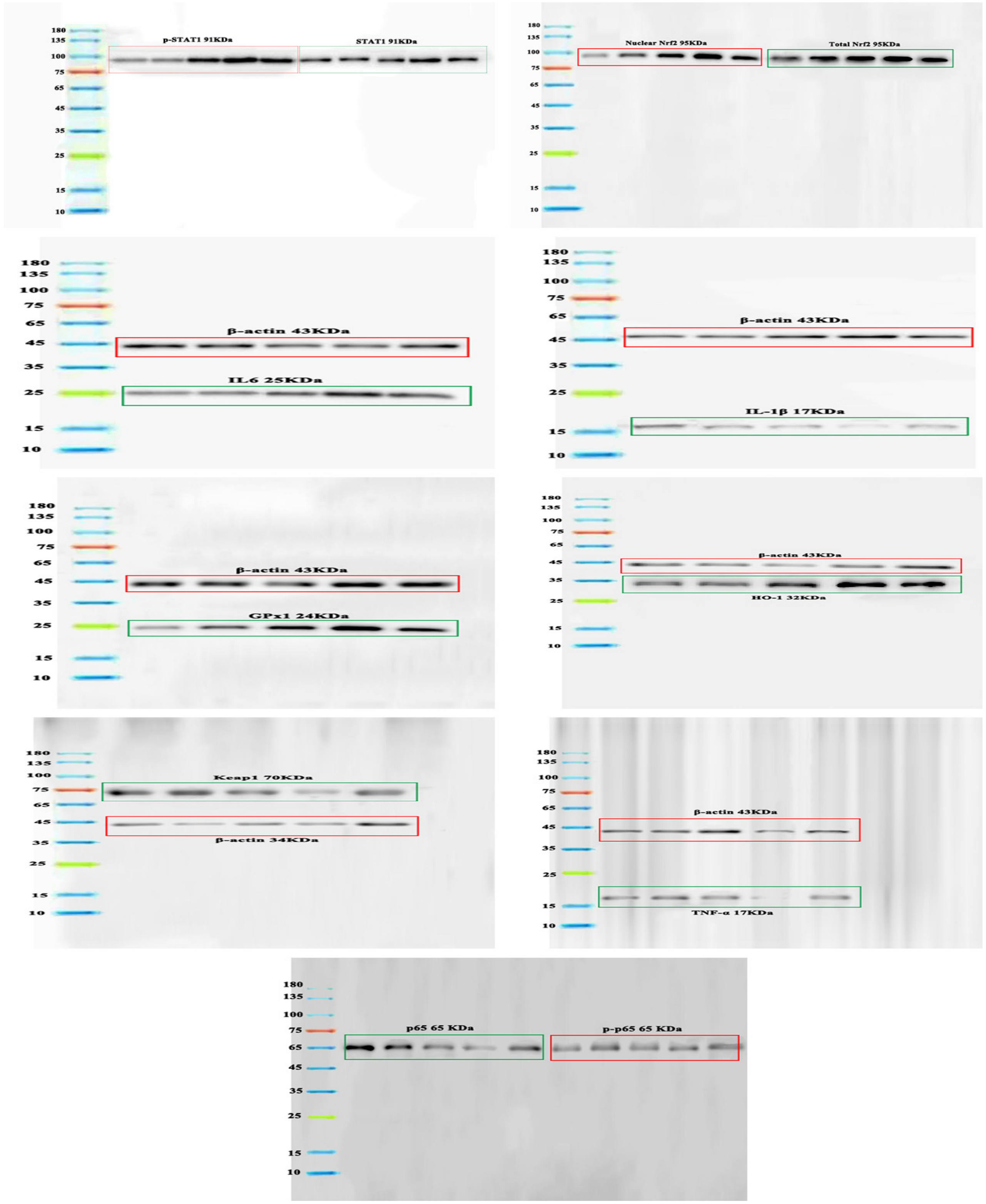

Figure S1. Representative original Western blot images showing the expression of p-STAT1, STAT1, nuclear Nrf2, total Nrf2, IL-6, IL-1β, GPx1, HO-1, Keap1, and TNF-α in rat liver tissues from fermented apple pomace-treated rats. β-actin was used as the loading control for total protein. Nuclear Nrf2 was normalized to total Nrf2. $n = 3$ independent biological replicates per treatment. Bands were quantified using ImageJ; blinding was not applied. Antibodies were used according to the manufacturers' instructions.

https://doi.org/10.3390/foods15213770

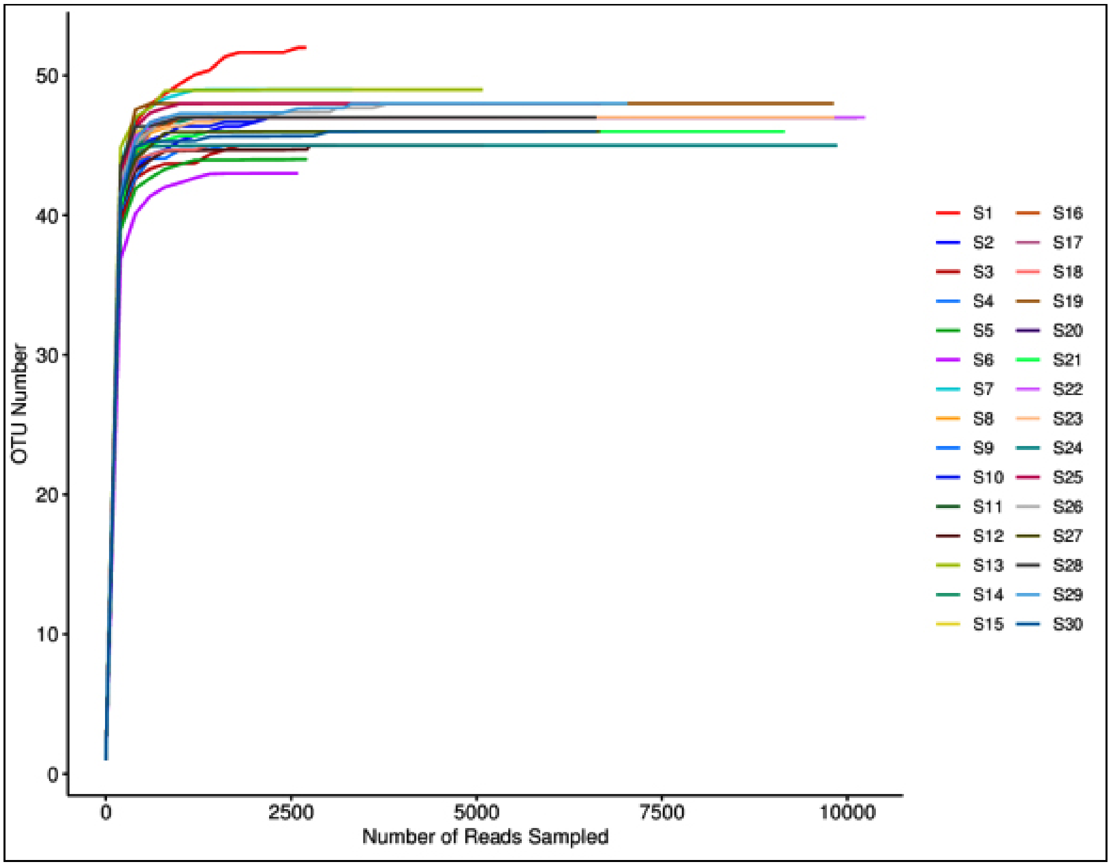

Figure S2. Rarefaction curves of the fecal microbiota in rats. The x-axis represents the number of reads sampled, and the y-axis represents the number of observed OTUs. Each curve represents one sample (S1–S30). The curves reached a plateau at approximately 2,500–5,000 reads, indicating that the sequencing depth was sufficient to capture the major microbial diversity. OTU, operational taxonomic unit.
